# Ancient hybridization and repetitive element proliferation in the evolutionary history of the monocot genus *Amomum* (Zingiberaceae)

**DOI:** 10.3389/fpls.2024.1324358

**Published:** 2024-04-19

**Authors:** Kristýna Hlavatá, Eliška Záveská, Jana Leong-Škorničková, Milan Pouch, Axel Dalberg Poulsen, Otakar Šída, Bijay Khadka, Terezie Mandáková, Tomáš Fér

**Affiliations:** ^1^ Department of Botany, Faculty of Science, Charles University, Prague, Czechia; ^2^ Institute of Botany, Czech Academy of Science, Průhonice, Czechia; ^3^ Herbarium, Singapore Botanic Gardens, National Parks Board, Singapore, Singapore; ^4^ Department of Biological Sciences, National University of Singapore, Singapore, Singapore; ^5^ Central European Institute of Technology, Masaryk University, Brno, Czechia; ^6^ National Center for Biomolecular Research (NCBR), Masaryk University, Kamenice, Czechia; ^7^ Tropical Diversity Section, Royal Botanic Garden Edinburgh, Edinburgh, United Kingdom; ^8^ Department of Botany, National Museum in Prague, Prague, Czechia; ^9^ Department of Experimental Biology, Faculty of Science, Masaryk University, Brno, Czechia

**Keywords:** genome evolution, genome size, interspecific hybridization, repetitive DNA, repeatome, phylogeny, 5S rDNA, Zingiberaceae

## Abstract

Genome size variation is a crucial aspect of plant evolution, influenced by a complex interplay of factors. Repetitive elements, which are fundamental components of genomic architecture, often play a role in genome expansion by selectively amplifying specific repeat motifs. This study focuses on *Amomum*, a genus in the ginger family (Zingiberaceae), known for its 4.4-fold variation in genome size. Using a robust methodology involving PhyloNet reconstruction, RepeatExplorer clustering, and repeat similarity-based phylogenetic network construction, we investigated the repeatome composition, analyzed repeat dynamics, and identified potential hybridization events within the genus. Our analysis confirmed the presence of four major infrageneric clades (A–D) within *Amomum*, with clades A–C exclusively comprising diploid species (2n = 48) and clade D encompassing both diploid and tetraploid species (2n = 48 and 96). We observed an increase in the repeat content within the genus, ranging from 84% to 89%, compared to outgroup species with 75% of the repeatome. The SIRE lineage of the *Ty1-Copia* repeat superfamily was prevalent in most analyzed ingroup genomes. We identified significant difference in repeatome structure between the basal *Amomum* clades (A, B, C) and the most diverged clade D. Our investigation revealed evidence of ancient hybridization events within *Amomum*, coinciding with a substantial proliferation of multiple repeat groups. This finding supports the hypothesis that ancient hybridization is a driving force in the genomic evolution of *Amomum*. Furthermore, we contextualize our findings within the broader context of genome size variations and repeatome dynamics observed across major monocot lineages. This study enhances our understanding of evolutionary processes within monocots by highlighting the crucial roles of repetitive elements in shaping genome size and suggesting the mechanisms that drive these changes.

## 1 Introduction

Genome size, also known as C-value or haploid nuclear DNA content (hereafter referred to as GS), is a fundamental parameter in the study of organismal evolution. In land plants, GS exhibits remarkable variation, spanning up to 2,400-fold (Pellicer et al., 2018). Both genome expansion and contraction have been recognized as major driving forces of diversification in land plants (Cheng et al., 2014; Meudt et al., 2015; Simonin and Roddy, 2018). Genome expansion, often linked to whole genome duplication events, has been a historical precursor to speciation and the emergence of novel morphological features in various plant lineages (Qiao et al., 2022). Another mechanism that is profoundly shaping GS is amplification of repetitive sequences, in which transposable elements play a pivotal role (Pulido and Casacuberta, 2023).

Repetitive elements, often referred to as “tuning knobs of evolution” (King et al., 1997; hereafter referred to as repeats), are integral components of plant genomes. They can constitute as little as 3% in *Utricularia gibba* or as much as 91% of the entire genome in *Allium sativum* (Sun et al., 2020). They play the key roles in gene expression regulation (Garrido-Ramos, 2012; Bennetzen and Wang, 2014) and can evolve into new genes due to their rapid evolutionary rates (Mehrotra and Goyal, 2014). From the evolutionary perspective, the proliferation of repeats has been associated with diversification of new phylogenetic groups (Gaiero et al., 2019; Hloušková et al., 2019) and facilitates adaptation to changing environments (Jansz, 2019; Kumar and Mohapatra, 2021). Given the linear relationship between repeat content and GS within specific ploidy level (Lee and Kim, 2014), it is plausible to hypothesize that factors influencing repeat amplification align with those governing changes in GS.

Interspecific hybridization, a widespread phenomenon in angiosperms (Mallet, 2005), may play an important role in GS increase triggered by so-called genomic shock after subgenome merger (O’Neill et al., 1998; Ungerer et al., 2006; Wei et al., 2021). However, hybridization events may also lead to repeat deactivation and genome downsizing (Renny-Byfield et al., 2013; Heyduk et al., 2021), through processes such as illegitimate recombination or unequal homologous recombination (Bennetzen et al., 2005; Staton et al., 2012). Recent advancements in phylogenetics and phylogenomics, such as target enrichment techniques (Cao et al., 2019), now enable the robust identification of hybrid species and lineages and explore the role of hybridization in GS changes.

The tropical genus *Amomum*, comprising approx. 64 species (de Boer et al., 2018), represents a distinctive case study group for exploring processes associated with GS amplification. *Amomum* exhibits the most significant GS variation within the entire family Zingiberaceae, ranging from 1,731 to 7,656 Mb, representing a 4.4-fold difference (Záveská et al., 2024). In *Amomum*, two tetraploid species are known (2n = 96), with GS values of 6,254 and 7,656 Mb (Hlavatá et al., 2023). The diploid species (2n = 48) display substantial GS variation, ranging from 1,731 to 4,699 Mb, representing a 2.7-fold difference. A well-supported phylogeny, constructed using Hyb-Seq based on 449 nuclear genes, identified four main clades (A, B, C, and D; Hlavatá et al., 2023) and increasing trend of GS along the phylogeny. Evidence of cyto-nuclear discordance, possibly indicating hybridization, was detected and warrants further investigation (Hlavatá et al., 2023). In this context, we hypothesize that the enlargement of GS in diploid *Amomum* species is a result of an expansion of repeats triggered by interspecific hybridization. Particularly, we aim to answer the following questions: i) what is the repeatome composition in the genus *Amomum*?, ii) does interspecific hybridization play a role in the evolution of *Amomum* and its repeatome?; and iii) is the evolution of repeats correlated with phylogenetic relationships in *Amomum*? To answer these questions, we use a wide range of analyses starting with a revision of GS variation within the genus based on 52 *Amomum* accessions (33 species), continuing with a phylogenetic network reconstruction of 30 *Amomum* species, complemented with a qualitative and quantitative analysis of repeats in a subset of 11 *Amomum* species.

## 2 Methods

### 2.1 Plant material

A total of 52 accessions, corresponding to 30 distinct *Amomum* species and encompassing the documented morphological, phylogenetic, and cytological spectrum of the genus (Hlavatá et al., 2023), were employed in the present study to analyze genome size (GS) data. Reticulate relationships were reconstructed for these 30 species, while a subset of 11 accessions (plus two outgroup species included for comparative purposes) was further designated for an in-depth examination of repeat content. The selection of these subsets was devised to ensure that they represented the following aspects i) the primary phylogenetic clades within the genus *Amomum*, ii) variability in GS within and among these clades and iii) variation in ploidy levels observed across the genus. A comprehensive listing of all samples and their characteristics is provided in **Supplementary Table 1**.

### 2.2 Genome size estimation and chromosome counts

Nuclear GS (referred to as nuclear DNA 1C values in Mb) data were sourced from our previous study (Hlavatá et al., 2023) for a total of 52 *Amomum* accessions plus 2 outgroup species. Chromosome numbers, and ploidy levels were also sourced from Hlavatá et al. (2023) for 12 *Amomum* accessions representing 12 species (**Supplementary Table 1**).

### 2.3 Sequencing data from target enrichment (Hyb-Seq) for species networks reconstruction

Raw data derived from Hyb-Seq encompassing 30 *Amomum* accessions were obtained from Hlavatá et al. (2023) and were processed similarly as in the previous study using HybPhyloMaker 1.6.4 (Fér and Schmickl, 2018) up to the reconstruction of gene trees based on total of 448 loci employing RAxML 8.2.4 (Stamatakis, 2014) with 1,000 standard bootstrap replicates and per exon partitioning. In cases where gene trees contained uncertain nodes with bootstrap support below 50, branches were collapsed. These gene trees were then employed in the reconstruction of species networks using a maximum pseudo-likelihood (MPL) framework function ‘InferNetwork_MPL’ (Yu and Nakhleh, 2015) and implemented in PhyloNet 3.6.1 (Than et al., 2008). Since the comprehensive exploration of dataset comprising 30 accessions with a larger number of reticulations (>2) utilizing a MPL approach is limited by prohibitive runtime costs (Than et al., 2008; Skopalíková et al., 2023) we adopted a sequential, stepwise approach for the analysis of our dataset. Initially, we constructed a species network for the entire dataset of 30 accessions, hereafter referred to as ‘*complete dataset*’, allowing for a maximum of two reticulations. Subsequently, we conducted a separate analysis on a subset comprising 17 accessions, which represented 16 species belonging to clade D, hereafter termed the *‘clade D dataset’*, again allowing for a maximum of two reticulations. Prior to the Phylonet analyses, the gene trees were rooted using Newick Utilities 1.6 (Junier and Zdobnov, 2010). For the *complete dataset*, *A. subulatum* and *A. petaloideum* served as rooting taxa, while *A.* aff. *curtisii*, *A. latiflorum* and *A. corrugatum* were employed for rooting the *clade D dataset.* Each analysis involved ten runs with default settings, resulting in the generation of five optimal networks per analysis. The selection of the best-fitting network was accomplished by applying the Akaike information criterion (AIC, AIC = 2*k - 2*L). Here, ‘k’ represented the number of parameters, which included the number of branches and the number of reticulations, while ‘L’ denoted the likelihood value (Keuler et al., 2020). To present the findings effectively, we combined the best-fitting models from both datasets into a unified phylogenetic network.

### 2.4 Low-coverage sequencing

Genome skimming was conducted on a subset of 13 accessions, consisting of 11 *Amomum* species and two closely related outgroup species (*Aframomum melegueta* and *Renealmia polypus*, **Supplementary Table 1**). DNA sequencing libraries were prepared as described in **Supplementary Methods M1** and subjected to sequencing on an Illumina NextSeq, utilizing a 300-cycle sequencing kit to generate 150 bp paired-end reads. The sequencing process was conducted at the Central European Institute of Technology (CEITEC), Masaryk University in Brno, Czech Republic. Raw reads resulting from this process were subsequently uploaded to the Sequence Read Archive (SRA) under the BioProject designation (ID PRJNA1029323).

### 2.5 Repeatome analysis

Read clustering and subsequent automated quantification of repetitive elements (repeats) were performed on the Galaxy platform (Afgan et al., 2018; https://repeatexplorer-elixir.cerit-sc.cz/) following the established protocol as described by Novák et al. (2020). Classification of repeats was carried out in accordance with the automatic procedure of RepeatExplorer (REXdb; Neumann et al., 2019) and was subject to manual verification. For repeat identification, BLAST (Altschul et al., 1990) was employed to search against a comprehensive repeat library compiled from various publicly accessible sources, including msRep (Liao et al., 2022; https://msrepdb.cbrc.kaust.edu.sa/), PlantRep (Luo et al., 2022; http://www.plantrep.cn/), RepeatMasker (Smit et al., 2013; https://www.repeatmasker.org/), and Musaceae-specific repeat database (Novák et al., 2014; https://olomouc.ueb.cas.cz/en/content/dna-repeats/). The manually reviewed file for each accession was subsequently utilized for the quantification of repeats, considering the known GS of the respective accession. This process was carried out using the script available at https://github.com/tomas-fer/scripts/blob/master/makeREsummary.sh. Barplots representing the main repeat groups were constructed using Microsoft Excel (Microsoft Corporation, 2018).

An additional analysis of tandem repeats was conducted using Tandem Repeat Analyzer (TAREAN; Novák et al., 2017) for all accessions. This analysis aimed to identify potential satellite sequences that may not have been detected by the RepeatExplorer analysis and to provide insights into the presence and organization of 5S rDNA clusters. In TAREAN, the cluster size threshold was established at 0.01, and the processing queue was configured for “extra long” run times to accommodate the analysis of the maximum feasible number of reads. In the context of 5S rDNA, diploid specimens typically exhibit as single-looped circular graphs, whereas accessions of hybrid and/or polyploid origin may present more complex multi-looped graphs, as detailed by Garcia et al. (2020). Hence, the examination of 5S rDNA can serve as an indicator of hybridization (Garcia et al., 2020).

### 2.6 Comparative analysis of repeats

A comparative analysis, involving the simultaneous clustering of reads from all accessions, was performed in RepeatExplorer. This analysis adhered to the established protocol of Novák et al. (2020) and employed default settings. A random subsample of 1,000,000 reads was selected from each accession for this analysis. From this analysis, the distribution of the 225 most prevalent comparative repeat clusters, hereafter referred to as “sub-lineages”, was graphically represented, excluding clusters originating from plastid-derived sequences. To integrate these repeat sub-lineage abundances onto the phylogeny, the “*contMap*” function from the “*phytools*” package within the R environment (Revell, 2012; R. 4.2.1, R Core Team, 2022) was employed.

### 2.7 Phylogenetic signal and correlation of repeat proportions

In the representation of phylogenetic relationships among the 13 accessions studied for repeats, the ASTRAL species tree, as constructed based on HybSeq data and presented in Hlavatá et al. (2023), served as the foundation. This tree was appropriately tailored by pruning using the “*drop.tip*” function in the “*ape*” package within the R environment to exclusively encompass the specific subset of sampled accessions. To assess the phylogenetic signal, represented as Pagel’s λ (Pagel, 1997, 1999), associated with the proportions of repeats (for lineages, superfamilies and groups), the “*phylosig*” functions within “*phytools*” package in R was employed. The degree of simple correlation (adjusted R-squared) between the quantity of repeats and 1C GS (both considering and not considering the phylogenetic context) was computed. This was achieved using the “*geiger*” (Pennell et al., 2014) and “*caper*” (Orme et al., 2018) packages in the R environment. The specific functions utilized for this purpose included “*comparative.data*”, “*model.pgls*”, and “*anova*”. These correlations were calculated for both the overarching repeat groups and individual repeat lineages.

### 2.8 Similarity based consensus network

The matrices, originally indicating the observed/expected number of edges between species as derived from *RepeatExplorer* clustering analysis, were transformed into distance matrices as described by Vitales et al. (2020). In this process, matrices that represented clusters without any edges connecting species were entirely excluded from the analysis. Additionally, both outgroup species were entirely omitted from consideration since they exhibited very few connections with the ingroup. For the construction of neighbor-joining trees, we employed the “*ape*” package (Paradis and Schliep, 2019) within the R environment. Furthermore, a consensus network was established using the *SplitsTree* (Huson and Bryant, 2006), based on the method by Holland and Moulton (2003). Only splits that garnered support in a minimum of 10% of the trees were taken into account for subsequent analysis.

### 2.9 Fluorescent *in situ* hybridization

Genomic DNA was extracted from silica-dried or freshly collected leaves of selected accessions using the NucleoSpin Plant II kit (Macherey-Nagel). The highly variable GAG domains of retrotransposable elements (REs) SIRE (*Ty1-Copia*) and Tekay (*Ty3-Gypsy*) were sequentially chosen for FISH probe. The details of probe design is given in **Supplementary Methods M2**.

Mitotic chromosomes were isolated from root tips of *A.* aff. *repoeense* (clade B, 2n = 48), *A.* aff. *curtisii* (clade D1, 2n = 48), *A. trilobum* (clade D3, 2n = 48), and *A. cinnamomeum* (clade D3, 2n = 96) according to Mandáková and Lysák (2016a). The preparation and labeling of DNA probes and FISH followed the published protocols (Mandáková and Lysák, 2016b).

### 2.10 Comparative analysis of the repeatome structure across monocots

To contextualize the repeatome structure of *Amomum* in a broader context, we conducted an extensive data collection exercise encompassing various genomic and repeatomic characteristics across diverse monocot genera. This endeavor leveraged previously published studies employing diverse methodologies. Our primary data source included plant genome information available up to September 2023 from https://www.plabipd.de. We employed this resource to gather a comprehensive array of genomic features, conduct a repeatome analysis, and facilitate comparison across 17 monocot families. In the pursuit of comprehensive data, we thoroughly examined documented repeats from over 150 monocot plant species with a particular focus on multiple publications available for individual species when accessible. Notably, several species featured multiple publications, such as *Musa acuminata* (see **Supplementary Table 4**). To establish comparison data, we exclusively considered articles that presented information on the various repeat superfamilies and transposable elements, particularly LTR/*Ty1-Copia* and LTR/*Ty3-Gypsy*, which were annotated, quantified, and expressed as percentages relative to the entire genome. Publications utilizing the RepeatExplorer pipeline were excluded, as this method diverges from the approaches employed in the majority of other selected papers. For studies lacking essential details necessary for comparison, we meticulously reviewed the findings, supplementary materials, and other available data. In some instances, we recalculated several repeat families based on the published data. For the purposes of comparison, we used the percentage of the entire repeat content of the particular genome, as well as the percentages of LTR/*Ty1-Copia* and LTR/*Ty3-Gypsy* elements, LTRs, LINEs and DNA transposons (**Supplementary Table 4**). Additionally, we extracted and log-transformed published GS for various monocot plant species and families from https://cvalues.science.kew.org/ for further analysis (**Supplementary Table 4**). For Zingiberaceae and its subfamilies, we used our own GS measurements (Záveská et al., 2024).

## 3 Results

### 3.1 Revealing hybridization events in the evolutionary history of *Amomum*

**Figure 1:**
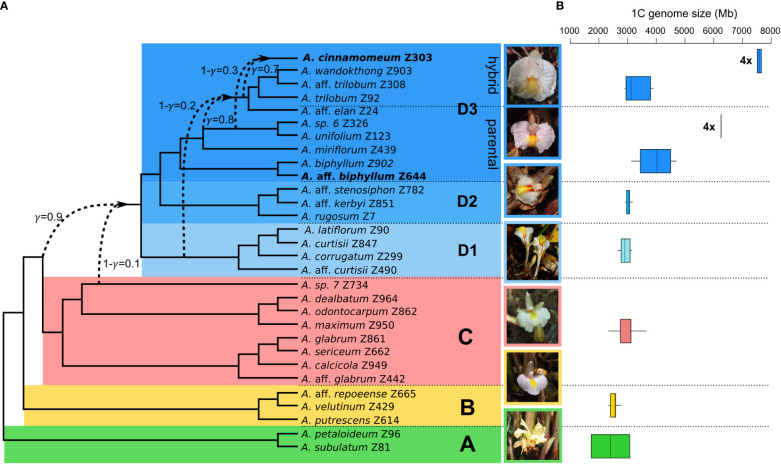
Phylogenetic network and genome size variation in the genus *Amomum*. **(A)** The phylogenetic network illustrates the interrelationships among 30 species in the genus *Amomum*. It was constructed based on optimal networks derived from PhyloNet maximum pseudo-likelihood analysis of both the ‘*complete dataset*’ and ‘*clade D dataset*’ (see Methods). All species are diploid (2n = 48), except for two tetraploids (2n = 96) highlighted in bold. The primary clades (A–D) and subclades (D1, D2, D3), as originally defined in Hlavatá et al. (2023), are visually distinguished through distinct color-coding. Dashed lines highlight instances of ancient hybridization predating diversification of clade D and within clade D, particularly in part of the D3 subclade (‘D3 hybrid’), as well as the recent hybrid origin of tetraploid *A*. *cinnamomeum*. The γ value signifies the probability of inheritance from one potential ancestor, while 1-γ represents inheritance from the second ancestor. Photographs showcasing the flowers of clade representatives are presented (clade A: *A*. *subulatum*, B: *A*. aff. *repoeense*, C: *A*. aff. *glabrum*, D1: *A*. aff. *curtisii*, D2: *A*. *rugosum*, and D3: *A*. *cinnamomeum*; photographed by J.L.-Š. and K.H.). **(B)** The analysis of genome size variation, as indicated by 1C values in Mb, within the examined clades. The data is drawn from 52 *Amomum* accessions (33 species). Notably, the genome size variations of the two tetraploid species *A.* aff. *biphyllum* and *A*. *cinnamomeum*) are presented in separate box plots within the ‘D3 parental’ and ‘D3 hybrid’ clades and denoted by ‘4x’ labels.

The PhyloNet analyses of the *‘complete dataset*’, comprising 30 *Amomum* species, consistently yielded the same topology with a single reticulation. The optimal model, determined by the highest log-probability and the lowest AIC score over multiple runs with varying priors on the number of reticulations, was obtained from a run specifying two predefined reticulations (**Table 1**; **Supplementary Figure 3**). The network’s topology closely aligns with the genus’s phylogeny as previously reported by Hlavatá et al. (2023), distinguishing four primary clades, denoted as A, B, C, and D, along with three subclades within clade D (D1–D3). Additionally, it introduces a reticulation indicating introgression from *Amomum* sp. 7 (1-γ=0.1, where γ represents the inheritance probability, Yu and Nakhleh, 2015) of clade C (or its ancestor) into ancestor of clade D. For the ‘*clade D dataset’*, the most suitable network also resulted from a run specifying two predefined reticulations (**Table 1**; **Supplementary Figure 4**). This network reveals i) introgression from the ancestor of D1 (1-γ = 0.2) into a specific lineage within subclade D3, here referred to as ‘D3 hybrid’; and ii) introgression from the ancestors of *Amomum* sp. 6 and *A*. *unifolium* within the D3 subclade (1-γ = 0.3) into the tetraploid *A*. *cinnamomeum*. The group of species that were not affected by hybridization and form a monophylum within the D3 subclade is further called the ‘D3 parental’ subclade. **Figure 1A** summarizes the outcomes of PhyloNet analyses on these two datasets, highlighting three significant hybridization events within the genus. As the hybridization events occurred prior to the diversification of specific groups (clade D and clade ‘D3 hybrid’), they align with the definition of ‘ancient hybridization’ proposed by Stull et al. (2023). Subsequently, in the following text, we also employ this term in the same context.

**Table 1:** PhyloNet outcomes and AIC assessments for the determination of the optimal network in the ‘*complete dataset*’ and ‘*clade D dataset*’.

	# reticulations	lnL	ΔlnL	# branch lengths	k	AIC	ΔAIC
*complete dataset*	0	-1374253.32	–	32.2	32.2	2748571.05	1069
	1	-1373843.13	410	35.6	36.6	2747757.46	255
	**2**	**-1373715.25**	**128**	**35.8**	**37.8**	**2747502.11**	**0**
*clade D*	0	-261422.00	–	18.8	18.8	522881.61	433
	1	-261291.72	130	21	22	522625.44	177
	**2**	**-261202.79**	**89**	**21.4**	**23.4**	**522448.37**	0

The optimal network is indicated in bold. In this context, ‘lnL’ denotes the likelihood value, while ‘k’ represents the cumulative count of reticulations and branch lengths, serving as the number of parameters involved in the AIC computation.

### 3.2 Genomic variation and repeat composition in *Amomum* and outgroup species

The GS (1C value) of *Amomum* species exhibited considerable variation, ranging from 1,731 Mb in *A. subulatum* to 7,656 Mb in *A. cinnamomeum* (**Figure 1B**), whereas the outgroup species displayed lower GS, with 1,006 Mb in *Aframomum melegueta* and 1,224 Mb in *Renealmia polypus*. Diploid *Amomum* species exhibited total repeat percentages ranging from 85% in *A. miriflorum* to 88% in *A. calcicola* and *A. subulatum* (**Supplementary Table 2**). The proportions of repeat content in tetraploids are well within the diploid range, with 87% in *A.* aff. *biphyllum* and *A. cinnamomeum*.

**Figure 2:**
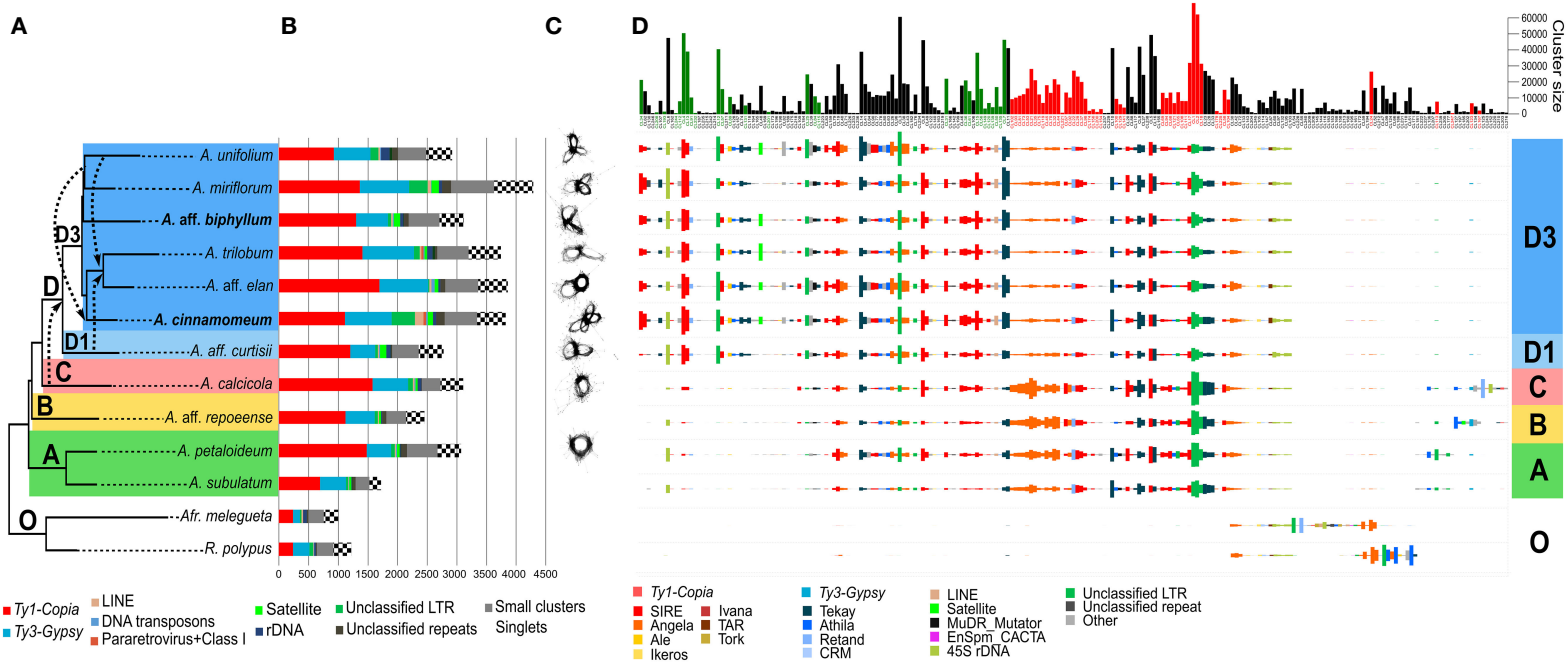
Comprehensive repeatome analysis in *Amomum* species. **(A)** A species tree constructed using Hyb-Seq data, encompassing eleven *Amomum* species and two outgroup species, which were subjects of repeatome exploration. Hybridization events, as revealed by PhyloNet analysis with a broader sampling, are represented by dashed arrows. The major phylogenetic clades **(A–D)** and subclades (D1 and D3), as originally characterized by Hlavatá et al. (2023), are indicated with discrete color-coding; “O” indicates the outgroup species. All species are diploid (2n = 48), except for two tetraploids (2n = 96) highlighted in bold. *A*. = *Amomum*, *Afr*. = *Aframomum*, *R.* = *Renealmia*. **(B)** Results from the RepeatExplorer clustering, quantified in Mb. The legend below the graph explains the repeat lineages. **(C)** Visualization of 5S rDNA clusters in individual species, illustrating increasing complexity in clade **(D)** The number of loops in 5S rDNA increases to two or more following ancient hybridization events. **(D)** Comparative analysis of repeats in *Amomum* species, adjusted to GS, conducted using RepeatExplorer. The graph illustrates the abundances of 225 repeat clusters (sub-lineages) in individual species. Size barplots of clusters displaying significant phylogenetic signals (Pagel’s λ = 1, *p < 0.05*) are color-coded as green (indicating amplification) and red (indicating reduction).

Among the 13 species analyzed by RepeatExplorer (**Figure 2**), the repeat composition of *Amomum* exhibited significant divergence from that of the outgroup (**Figures 2B, D**). Several sub-lineages, prevalent in *Amomum*, either underwent substantial amplification or emerged anew within the genus. In the majority of *Amomum* genomes, a significant portion was found to be dominated by LTR retrotransposons of the *Ty1-Copia* superfamily, with *Ty3-Gypsy* lineages representing the second most prevalent element. Unclassified LTRs constituted a substantial portion of the genome in certain species, particularly in the tetraploid *A. cinnamomeum* and diploid *A. miriflorum*. Tandem repeats were more abundant in some species (*A.* aff. *curtisii*, *A*. aff. *biphyllum*, *A. elan*) while their proportion remained notably low in others (*A. subulatum*, *A.* aff. *repoeense*, *A. unifolium*). *A. cinnamomeum* and *A. miriflorum* moreover exhibited a relatively high proportion of unclassified repeats. Single-copy genome content and “small clusters” (those comprising less than 0.01% of reads from the dataset) constituted substantial portions of the genome; nevertheless, their proportions displayed minimal variability among species. For detailed quantification data, see **Supplementary Table 2**.

### 3.3 Comparative analysis reveals genomic distinctions and repeat composition in *Amomum*

The comparative analysis (**Figure 2D**) unveiled stark disparities between the outgroup and ingroup, as well as variations among individual clades within the genus *Amomum*. Most repeat sub-lineages shared with the outgroup showed a reduction in *Amomum*, while few experienced amplification. Notably, certain lineages such as Angela (*Ty1-Copia*) or Athila (*Ty3-Gypsy*) exhibited different sub-lineage compositions in the outgroup compared to *Amomum*. *Amomum* featured several sub-lineages of unclassified LTR repeats not present in the outgroup. Clades A, B and C within *Amomum* exhibited highly similar repeat compositions, with minor distinctions in less abundant repeats, such as Athila and Retand (*Ty3-Gypsy*). In contrast, clade D showcased the emergence of a new, abundant, unclassified LTR sub-lineage, along with reductions in several other sub-lineages within this clade. Notably, clade D exhibited a pronounced amplification of numerous sub-lineages, including those of SIRE (*Ty1-Copia*), Tekay (*Ty3-Gypsy*), 45S rDNA, and unidentified LTRs, while experiencing reductions in other sub-lineages, particularly within Angela, and to a lesser extent, some SIRE and unidentified LTR sub-lineages. The distinctions in repeatome between subclades D1 (represented solely by *A.* aff. *curtisii*) and D3 were relatively minor, except for the notable amplification of specific SIRE and Tekay sub-lineages in subclade D3. Within subclade D3, unclassified LTRs and unclassified repeats seemed to contribute to the observed increase in GS in select taxa, such as *A. miriflorum* and tetraploid *A. cinnamomeum*. Chromosome localization of SIRE and Tekay in *A.* aff. *repoeense* (clade B, 2n = 48), *A.* aff. *curtisii* (clade D1, 2n = 48), *A. trilobum* (clade D3, 2n = 48), and *A. cinnamomeum* (clade D3, 2n = 96) confirmed the abundant presence of both elements in the genomes. Their distribution appeared evenly dispersed across chromosomes of all species (**Supplementary Figure 5**).

The 45S rDNA displayed variable amplification within certain species in clade D, with larger genome sizes such as in *A. unifolium* and *A. trilobum*, but lesser amplification in others such as *A. miriflorum* and *A.* aff. *elan*. Surprisingly, *A.* aff. *curtisii*, despite having a smaller genome, exhibited notable 45S rDNA amplification (**Figures 2B, D**). Ribosomal DNA content varied across the genus, with the smallest amount observed in clade A.

From satellite regions a prominent cluster 48, was shared by most species in the D3 subclade, while a less abundant cluster 185 was exclusively found in *A. repoeense* of clade B (**Supplementary Figure 6**). While in the comparative analysis some species (e.g., *A.* aff. *curtisii*, *A. miriflorum*) showed no satellite presence, in a dedicated analysis employing TAREAN additional satellites were identified, although none appeared to be shared among different *Amomum* species. Notably, the clusters of 5S rDNA, analyzed in relation to phylogeny and hybridization estimates (**Figures 2A, C**), exhibited increased complexity. In clades A and C, 5S rDNA clusters exhibited a one-looped configuration, whereas in subclade D1 the number of loops increased to two after a presumed ancient hybridization event. Within subclade D3, two and more loops were observed, and the maximum number of loops reached four in the tetraploid *A. cinnamomeum* (**Figure 2B**).

### 3.4 Assessing phylogenetic signal in repeat content and its correlations with GS

We conducted a comprehensive examination of the phylogenetic signal at various levels, encompassing overall repeat content, superfamilies, and specific lineages. The overall repeat content as well as the content of *Ty3-Gypsy* (*p < 0.05*) demonstrated significant phylogenetic signal (**Supplementary Table 3**, *p < 0.05*). On the level of repeat lineages we identified significant phylogenetic signals in the quantities of Ale (*p < 0.01*) and Ivana (*p < 0.05*) (both *Ty1-Copia)* and Tekay (*Ty3-Gypsy*, *p < 0.05*). Among the total 225 repeat clusters corresponding to sub-lineages in the comparative analysis, 75 (33.3%) displayed significant phylogenetic signals, as indicated by the presence of red and green bars in the barplot shown in **Figure 2D**. Of these, 12.4% clusters displayed an increasing trend, while 20.9% exhibited a decreasing trend from clade A towards clade D. The remaining 76.4% of clusters did not demonstrate any significant phylogenetic signals (**Supplementary Table 3**). Diverse trends were observed within specific lineages, exemplified by the SIRE lineage, where 10 sub-lineages displayed an increase, while 12 showed a decrease (**Figure 2D**; **Supplementary Figure 7**). In certain lineages, all sub-lineages carrying phylogenetically significant signals displayed an increasing trend from clade A to subclade D3 (e.g., Tekay and LINEs; **Supplementary Table 3**). Conversely, all 17 sub-lineages within Angela and both Athila sub-lineages, which conveyed phylogenetic signals, demonstrated a decreasing trend along the phylogeny.

Phylogenetically adjusted correlation tests were performed to assess the relationship between GS and the total amount of repeats, repeat superfamilies and lineages. Notably, a significant correlation was observed between GS and the overall quantity of repeats. Furthermore, significant positive correlations were found between GS and the repeat quantities at the superfamily level for, *Ty1-Copia, Ty3-Gypsy* and *LINEs* (**Supplementary Table 3**). Similarly, positive correlations were shown for multiple lineages within above mentioned superfamilies, for satellites, for the group of pararetrovirus and unclassified Class I repeats, and for a group of unclassified repeats.

### 3.5 Consensus network analysis

**Figure 3:**
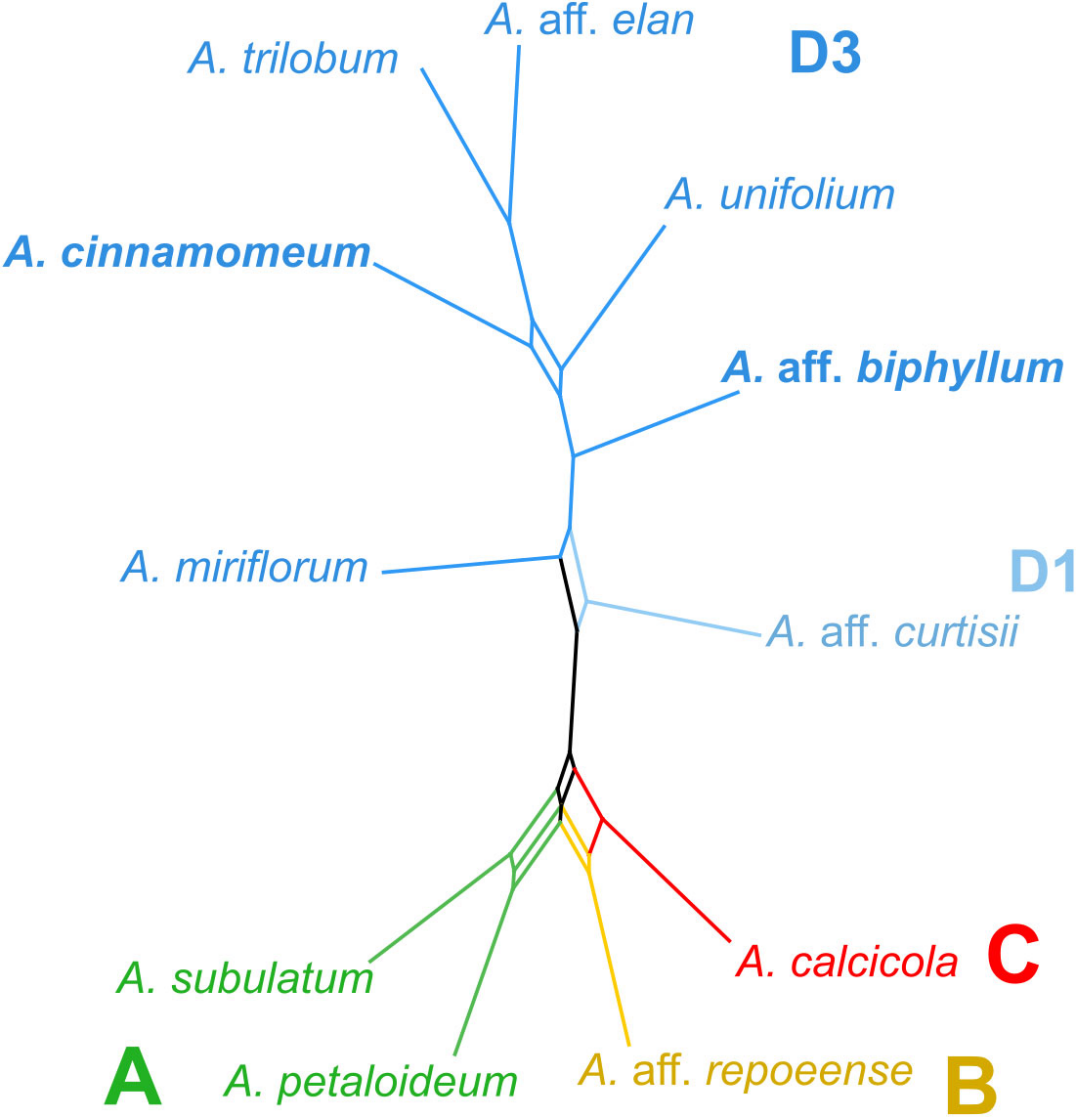
Repeat similarity network in *Amomum* species. A repeat similarity network was constructed based on 179 similarity matrices derived from repeats. The network represents the primary clades (A–D) and subclades (D1 and D3), as originally defined in Hlavatá et al. (2023), using distinct color-coding. All species are diploid (2n = 48), except for the two tetraploids (2n = 96), highlighted in bold. Clades are delineated by colors and letters. *A., Amomum*.

In the creation of a consensus network, utilizing 179 matrices representing the observed/expected number of edges between species from the RepeatExplorer clustering analysis (**Figure 3**), distinct patterns emerged. Specifically, two accessions from clade A exhibited close clustering within the network, while accessions representing clades B and C similarly formed a consolidated cluster. Accessions originating from subclade D3 constituted a distinctive cluster, adjacent to the accession representing subclade D1. Notably, the consensus network, constructed based on cluster similarity, demonstrated a remarkable alignment with the nuclear-gene based phylogeny, exhibiting congruence across all major clades (**Figure 3**).

### 3.6 Genome size and repeatome structure across monocot families

**Figure 4:**
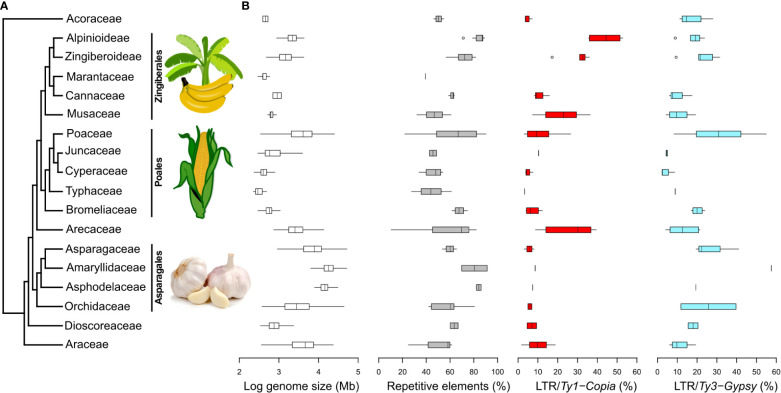
Phylogenetic relationships among 17 monocot plant families and selected genomic characteristics. **(A)** Monocot phylogenetic relationships based on APG IV. Images sourced from Wikimedia Commons. **(B)** Logarithm of GS, the percentages of overall repeat content, and the representation of *Ty1-Copia* and *Ty3-Gypsy* superfamilies in individual monocot families. These data were extracted from the Plant DNA C-values Database (https://cvalues.science.kew.org/) and selected genomic studies listed in **Supplementary Table 4**. Additional repeat lineages are presented in **Supplementary Figure 7**.

Within the monocots compared in this study, the range of GS varies from 196 Mb (as observed in *Amorphophallus rivieri*; Zhang et al., 2013) to 80,343 Mb (as evidenced in *Galanthus lagodechianus*; Zonneveld et al., 2003), i.e., 2–5 on log GS scale (**Figure 4B**). Notably, among the subset of monocots examined in our comparison, GS ranges exhibit considerable diversity, with the most pronounced variations occurring within the Poaceae (Poales) and Asparagaceae (Asparagales) families. The highest absolute GS values are encountered in families belonging to Asparagales.

In our comparison of repeatomes in monocots (**Figure 4B**), we observe the lowest proportion of repeats among monocots (10.5%) in *Korthalsia laciniosa* (Arecaceae; Ghosh Dasgupta et al., 2021), while the highest proportion (91.3%) is observed in *Allium sativum* (Amaryllidaceae; Sun et al., 2020). While garlic displays the most elevated proportion of repeats within the realm of monocots, the average repeat percentage within the Amaryllidaceae family ranks third, trailing behind the Asphodelaceae and Alpinioideae (Zingiberaceae).

The overall pattern of repeat proportion in monocot genomes is inherently reflected in the distribution of LTR proportions. While relatively less data is available for the proportion of LINEs, it is generally observed that their proportions are lower, with the maximum proportion reaching less than 20% of the genome. Notably, the pattern of LINE proportions closely aligns with that of *Ty3-Gypsy* proportions, with a conspicuous divergence observed in Poaceae genomes, where both the proportion and range of LINEs are notably lower than those of *Ty3-Gyps*y (**Supplementary Figure 8**).

Regarding DNA transposons, which have been more extensively documented, they are generally observed in relatively low proportions in most monocot families, typically within the range of up to 20% of the genome. Notably, the Cyperaceae genomes exhibit the highest proportion of DNA transposons, while the Bromeliaceae genomes showcase the most extensive range of proportions (**Supplementary Figure 8**).

The broadest ranges of repeatome proportions are observed in Poaceae and Arecaceae genomes. The distribution of *Ty1-Copia* and *Ty3-Gypsy* superfamilies varies among different monocot groups, with Arecaceae, Juncaceae, Musaceae, and Zingiberaceae displaying higher proportions of *Ty1-Copia*, while other families exhibit higher proportions of *Ty3-Gypsy*. The most significant quantities of *Ty1-Copia* are found in the family Zingiberaceae, while genomes with the highest *Ty3-Gypsy* proportions are identified in Poaceae.

Arecaceae, Poaceae, and Musaceae appear to exhibit broader ranges of both *Ty1-Copia* and *Ty3-Gypsy* percentages in comparison to other monocot groups, although this observation may partly arise from the limited datasets available for some of these groups. Poaceae and Orchidaceae stand out with the widest ranges of *Ty3-Gypsy* proportions.

## 4 Discussion

### 4.1 Repeatome proportion in *Amomum* genome is among the largest within monocots

Our comprehensive analysis of repeat proportions across monocot families shows that *Amomum*’s repeat content (85–88%) stands out for its exceptional richness, rivaling representatives from Amaryllidaceae, such as *Allium sativum*, which exhibits one of the highest recorded repeat percentages at 91% (Sun et al., 2020). It’s worth emphasizing that *Allium*’s genome is notably larger than other Amaryllidaceae species, as well as those of *Amomum*, with a 1C value of 15,844 Mb.

In contrast to Amaryllidaceae and broader groups such as Asparagales and Poales, where the *Ty3-Gypsy* superfamily predominates the repeatome, the *Ty1-Copia* superfamily takes precedence in shaping the repeatome in Alpinioideae, including *Amomum*. This predominance of *Ty1-Copia*, observed in our comparative analysis, is also noted in closely related Musaceae (e.g., Novák et al., 2014) and distantly related Arecaceae, suggesting a relatively rare pattern within monocots. The reasons for the abundance of one superfamily over the other in the genome are not fully understood due to the lack of literature on differences in functionality between *Ty1-Copia* and *Ty3-Gypsy*. It is speculated that this abundance may result from a combination of stochastic processes, selection, and phylogenetic relatedness (Venner et al., 2009).

Analysis of sequenced genomes of *Musa acuminata* (D’Hont et al., 2012) and *Areca catechu* (Yang et al., 2021) reveals that *Ty1-Copia* elements are concentrated along the centromeric regions. However, genomic studies on Zingiberaceae typically summarize only distribution densities of LTR elements along the genomes, showing either an even distribution of repeats along the chromosomes (e.g., in *Zingiber officinale*, Li et al., 2021) or a decreased concentration of repeats in regions with higher gene densities (e.g., *Curcuma longa*, Yin et al., 2022; *Wurfbainia villosa*, Yang et al., 2022). Comparisons of *Ty1-Copia* distribution between species indicate substantial differences, suggesting variation in plant genome structure (Heslop-Harrison et al., 1997). Our FISH results suggest that repeats in *Amomum* are also evenly distributed. Further detailed studies at the whole-genome level are necessary to better understand the role of *Ty1-Copia* in evolution.

### 4.2 The impact of repeat dynamics on genome size and evolution of *Amomum*

In our model plant genus *Amomum*, we observed a continuous increase in GS along the phylogeny, attributed to the amplification of *Ty1-Copia* elements, particularly the SIRE lineage. Similar patterns have been observed in the closely related Musaceae family (Novák et al., 2014) and the grass subtribe Loliinae (Moreno-Aguilar et al., 2022). Interestingly, the Angela sublineage (*Ty1-Copia*), often associated with significant GS amplification events in other species (e.g., in *Heloniopsis*, Pellicer et al., 2021, or *Passiflora*, Sader et al., 2021), showed a decreasing trend in *Amomum*, similar to observations in Musaceae (Novák et al., 2014). Our findings support previous suggestions that analyses at the sub-lineage level can elucidate the intricate mechanisms driving GS changes (e.g., Suguiyama et al., 2019).

To comprehend why GS increase in some groups while being constrained in others, Schley et al. (2022) investigated repeat-associated genome size expansion in light of ecological correlations. Their findings revealed that water stress inhibits repeat expansion through selection on upper genome size limits. This study builds upon previous suggestions that genome size may impact fitness, with larger genomes offering advantages in certain environments but disadvantages in others (Knight et al., 2005; Faizullah et al., 2021). Robust sampling is crucial to test such hypotheses, as demonstrated by Trávníček et al. (2019) and Carta and Peruzzi (2016). Within the Zingiberaceae family, Záveská et al. (2024) tested the hypothesis of large genome constraint and found that within the subfamily Alpinioideae, to which the genus *Amomum* belongs, plants with larger genomes thrive in shady habitats due to larger stomatal cells and more efficient photosynthesis (Beaulieu et al., 2008; Hodgson et al., 2010). Light intensity acts as a stressor in these habitats, where selection favors plants with smaller genomes (Knight et al., 2005). In *Amomum*, we have demonstrated that genome size is primarily shaped by repeat dynamics. The next step is to conduct explicit tests on the correlation of particular sub-lineages with various ecological and climatic factors to determine which sub-lineages are directly responsive to environmental stresses. From our current results, we speculate that *Amomum* species from clade D, possessing the largest genomes in Zingiberaceae, are well adapted to shady habitats, while those from basal groups have adapted to cope with light stress by maintaining smaller genomes.

### 4.3 Hybridization as a potential trigger of repeat amplification

Our detailed examination of the repeatome composition within clade D revealed the most substantial increase in multiple repeat lineages (**Figure 2D**). Strikingly, clade D was also identified as having an ancient hybrid origin, as previously suggested by cyto-nuclear discordance observed in Hlavatá et al. (2023). While causality cannot be definitively proven, it is plausible that the significant increase in multiple repeat lineages within clade D is closely associated with its hybrid lineage origin. Another clue to this hypothesis comes from the pattern of 5S rDNA clustering. Cluster graphs of species within early derived clades, such as A and C, displayed a single loop of 5S rDNA, consistent with non-hybridogenous species (Garcia et al., 2020). In contrast, the analyzed species from clade D exhibited two or more loops, suggesting at least one hybridization event at the base of clade D, and potentially more within the diversification of subclade D3. Notably, tetraploid *A. cinnamomeum* displayed four loops of 5S rDNA, indicating recent (allopolyploid) as well as ancient hybridization events.

Genomic shock occurs when a significant portion of the repeatome is reactivated or activated anew following hybridization. This phenomenon is often triggered by the merging of sub-genomes, leading to DNA demethylation and the activation of previously silenced repeats (Michalak, 2009; de Tomás and Vicient, 2024). Reactivation of a broad spectrum of repeats post-hybridization typically arises from the breakdown or malfunction of regulatory mechanisms (e.g., Shan et al., 2005; Ungerer et al., 2006; Wei et al., 2021), conferring potential benefits in terms of adaptability (Schrader and Schmitz, 2019). However, this proliferation usually exhibits a finite duration, as transposable elements tend to amplify and diversify within the new genome until either losing the ability to transpose or reactivating the silencing mechanism (Liu et al., 2022).

In various studies, hybridization events have been associated with a burst of specific repeat lineages. Examples include the activation of the *Gorge3* element in *Gossypiu*m (Hawkins et al., 2009), chromovirus-like retro elements in *Nicotiana* (Renny-Byfield et al., 2013), two *Gypsy*-like retrotransposons in *Phalaenopsis* (Hsu et al., 2020), or one satellite in *Spartina* (Giraud et al., 2021). Our analysis of *Amomum* revealed a significant amplification of 12.4% of sub-lineages in clade D compared to other clades, namely SIRE sub-lineages from the *Ty1-Copia* superfamily.

### 4.4 Exploring the utility of repeats as molecular markers and phylogenetic tools in *Amomum*

Repeats have proven to be valuable resources in molecular biology and phylogenetics as species-specific or group-specific markers (Rebollo et al., 2010). In the Musaceae, repeats have served as effective molecular markers, as the proliferation of certain groups often accompanied speciation (Novák et al., 2014).

To address insufficient resolution when using other markers, Dodsworth et al. (2017) proposed the utilization of repeats as molecular markers. Vitales et al. (2020) recently reconstructed phylogenetic relationships by employing matrices of similarity between repeat clusters. In *Amomum*, despite the smaller sample size, the repeat-based method provided congruent results, affirming that cluster similarities within *Amomum* can be effectively used to estimate phylogeny or complement other phylogenetic markers. Furthermore, our phylogenetic network analysis provided compelling support for the hybrid origin of the D3 ‘hybrid’ subclade, given its most distinct position within the network. This corroborates previous evidence indicating the hybrid origin of clade D3, strengthening our understanding of *Amomum*’s evolutionary history. On the other hand, we noted that this repeat-based phylogenetic method exhibited higher proportions of uncertainties in the relationships between clades A, B, and C (**Figure 4**), which mirrors the topological incongruences observed in previous studies involving chloroplast DNA, ribosomal DNA, and nuclear DNA (Hlavatá et al., 2023). These findings suggest that while the repeat-based phylogenetic approach is promising for resolving shallower evolutionary events, it may encounter limitations when addressing deeper phylogenetic relationships (Moreno-Aguilar et al., 2022).

## 5 Conclusions

In contrast to the predominant presence of *Ty3-Gypsy* elements in most monocot plant families, our study reveals that in *Amomum* (Zingiberaceae) and two other monocot families (Musaceae, Arecaceae), *Ty1-Copia* elements are the prevailing component of the repeatome. While explaining why *Ty1-Copia* elements prevail over *Ty3-Gypsy* elements in *Amomum* poses a challenge, we propose that the genome’s overall composition and the absolute amounts of repeats influence plant evolution by affecting cell size and photosynthetic efficiency, thereby impacting environmental stress tolerance. Specifically, we suggest that species with smaller genomes (from basal clades) may have faced selection pressure due to their distribution in more stressful (sunny) habitats, while species with larger genomes, facilitated by repeat amplification, are well adapted to shady habitats where selection against larger genomes is less stringent. In conclusion, the increase in genome size within terminal group D of the genus *Amomum* was likely triggered by ancient hybridization events, stimulating the amplification and diversification of various sub-lineages of both *Ty1-Copia* and *Ty3-Gypsy* subfamilies.

## Data availability statement

The data generated in the study are deposited in the GenBank Short Reads Archive, accession number PRJNA1029323.

## Author contributions

KH: Writing – review & editing, Writing – original draft, Methodology, Investigation, Data curation. EZ: Writing – review & editing, Writing – original draft, Methodology, Investigation, Data curation. JL-Š: Writing – review & editing, Resources. AP: Writing – review & editing, Resources. OŠ: Writing – review & editing, Resources. BK: Writing – review & editing, Investigation. TM: Writing – review & editing, Writing – original draft, Validation, Supervision, Funding acquisition, Conceptualization, Investigation. TF: Writing – review & editing, Supervision, Investigation, Conceptualization. MP: Investigation, Writing – review & editing.
