# Supplementary material for: Ancient hybridization and repetitive element proliferation in the evolutionary history of the monocot genus Amomum (Zingiberaceae)
Source: Front Plant Sci. 2024 Apr 19;15:1324358. doi: 10.3389/fpls.2024.1324358 (PMC11066291; doi:10.3389/fpls.2024.1324358)
Supplement: Supplementary file 1 [file DataSheet_1.docx]

Supplementary Material

**Ancient Hybridization and Repetitive Element Proliferation in the evolutionary history of the Monocot Genus *Amomum* (Zingiberaceae)**

**Kristýna Hlavatá, Eliška Záveská, Jana Leong-Škorničková, Milan Pouch, Axel Dalberg Poulsen, Otakar Šída, Bijay Khadka, Terezie Mandáková^*^ and Tomáš Fér**

*** Correspondence:** Terezie Mandáková
[terezie.mandakova@ceitec.muni.cz](mailto:terezie.mandakova@ceitec.muni.cz)

## 1 Supplementary Texts

## Supplementary Methods M1. Preparation of DNA library for low-coverage sequencing

DNA was sonicated to yield fragments 500-600 bp long, using a M220 Focused-ultrasonicator™ (Covaris). Verification of fragment length was accomplished through gel electrophoresis, with O'GeneRuler™ 100bp DNA Ladder Plus (Thermo Fisher Scientific) and Quick-Load® 1 kb DNA Ladder (New England BioLabs) employed as reference standards. Subsequently, libraries were prepared utilizing the NEBNext Ultra II DNA Library Prep Kit for Illumina (New England BioLabs). After library preparation, purification was carried out using QIAquick PCR Purification Kit (Qiagen), with the resulting DNA dissolved in 30 µl ddH2O. The quality and integrity of the DNA were assessed through gel electrophoresis, with O'GeneRuler™ 100bp DNA Ladder Plus (Thermo Fisher Scientific) and Quick-Load® 1 kb DNA Ladder (New England BioLabs) as reference markers. DNA fragments falling within the 500-600 bp range were excised from the gel, subjected to purification using QIAquick Gel Extraction Kit (Qiagen), and eluted into 20 µl ddH2O. Subsequent to gel extraction, the DNA products underwent PCR amplification and indexing. This was achieved using Q5 Hot Start HiFi PCR Master Mix (New England BioLabs) and NEBNext Multiplex Oligos for Illumina index primers (96 Unique Dual Index Primer Pairs, E6440S). Following amplification and indexing, the samples were purified twice using an Agencourt SPRI kit (Beckman Coulter), maintaining a kit-to-DNA ratio of 0.75:1, and were subsequently verified by gel electrophoresis. To determine the concentration of the samples, a Qubit® 2.0 fluorometer (Invitrogen) was employed, ensuring equimolar proportions.

**Supplementary Methods M2.** Design and preparation of probes for FISH

Firstly, multiple sequence DNA alignments of GAG domains were performed using MAFFT v7.490 (Katoh and Standley, 2013) implemented in Geneious Prime 2022.1.1 (https://www.geneious.com). Subsequently, distance tables showing pairwise % identities of sequences were generated. Maximum Likelihood (ML) phylogenetic trees for the two selected elements were inferred using IQ-TREE v2.2.0 (Nguyen et al., 2015; Hoang et al., 2018) with 1000 ultrafast bootstrap (UF bootstrap) replicates. The ML trees were manipulated and graphically modified in FigTree v1.4.4 (http://tree.bio.ed.ac.uk/software/figtree/). In both trees, main clades were defined as clusters of sequences with a sequence identity greater than 80% (**Supplementary Figure 1**, **Supplementary Figure 2**). Two predominant clades were chosen from each tree (SIRE_I, SIRE_VI, Tekay_I, Tekay_III). The subsequent steps included the design of probes tailored specifically to each selected clade. The details of the probes utilized for targeting GAG domains of SIRE and Tekay elements are provided in **Supplementary Table 5**. PCR primers were designed to GAG domains to obtain theoretical amplicons longer than 200 bp using Primer3 v2.3.7 implemented in Geneious Prime. The PCR amplification consisted of 1 cycle (95˚C for 5 min), 35 cycles (95˚C for 20s, 58˚C for 20s, and 72˚C for 20s), and 1 cycle (72˚C for 5 min). PCR products were purified using the NucleoSpin Gel and PCR Clean-up kit (Macherey-Nagel).

**References**

Katoh, K., and Standley, D. M. (2013). MAFFT multiple sequence alignment software version 7: improvements in performance and usability. *Mol. Biol. Evol.* 30, 772–780. doi: 10.1093/molbev/mst010.

Hoang, D. T., Chernomor, O., von Haeseler, A., Minh, B. Q., and Vinh, L. S. (2018). UFBoot2: Improving the Ultrafast Bootstrap Approximation. *Mol. Biol. Evol.* 35, 518–522. doi: 10.1093/molbev/msx281.

Nguyen, L.-T., Schmidt, H. A., von Haeseler, A., and Minh, B. Q. (2015). IQ-TREE: A Fast and Effective Stochastic Algorithm for Estimating Maximum-Likelihood Phylogenies. *Mol. Biol. Evol.* 32, 268–274. doi: 10.1093/molbev/msu300.

## 2 Supplementary Figures


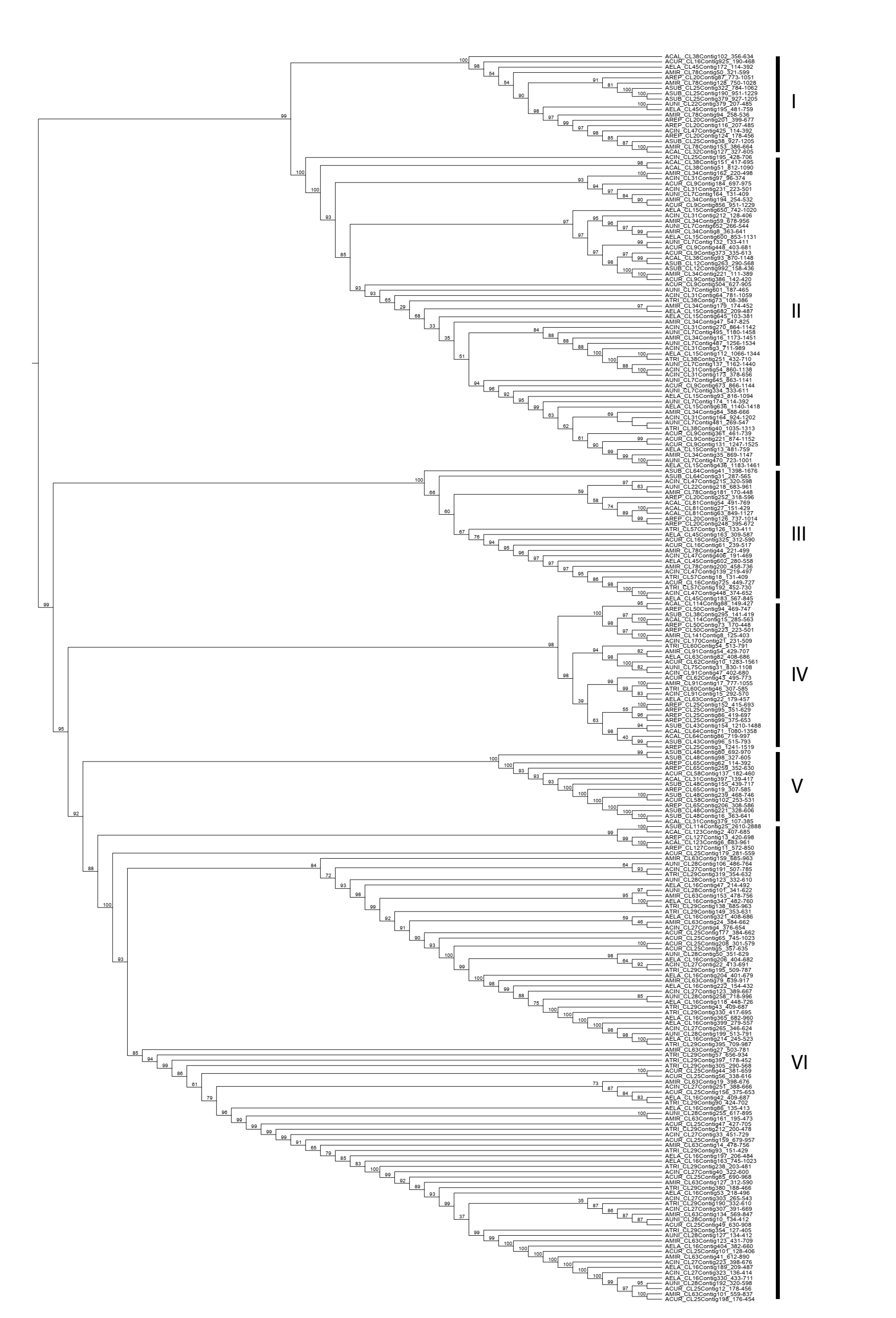


**Supplementary Figure 1.** Maximum likelihood phylogeny derived from the GAG domain of the SIRE element across selected *Amomum* species.


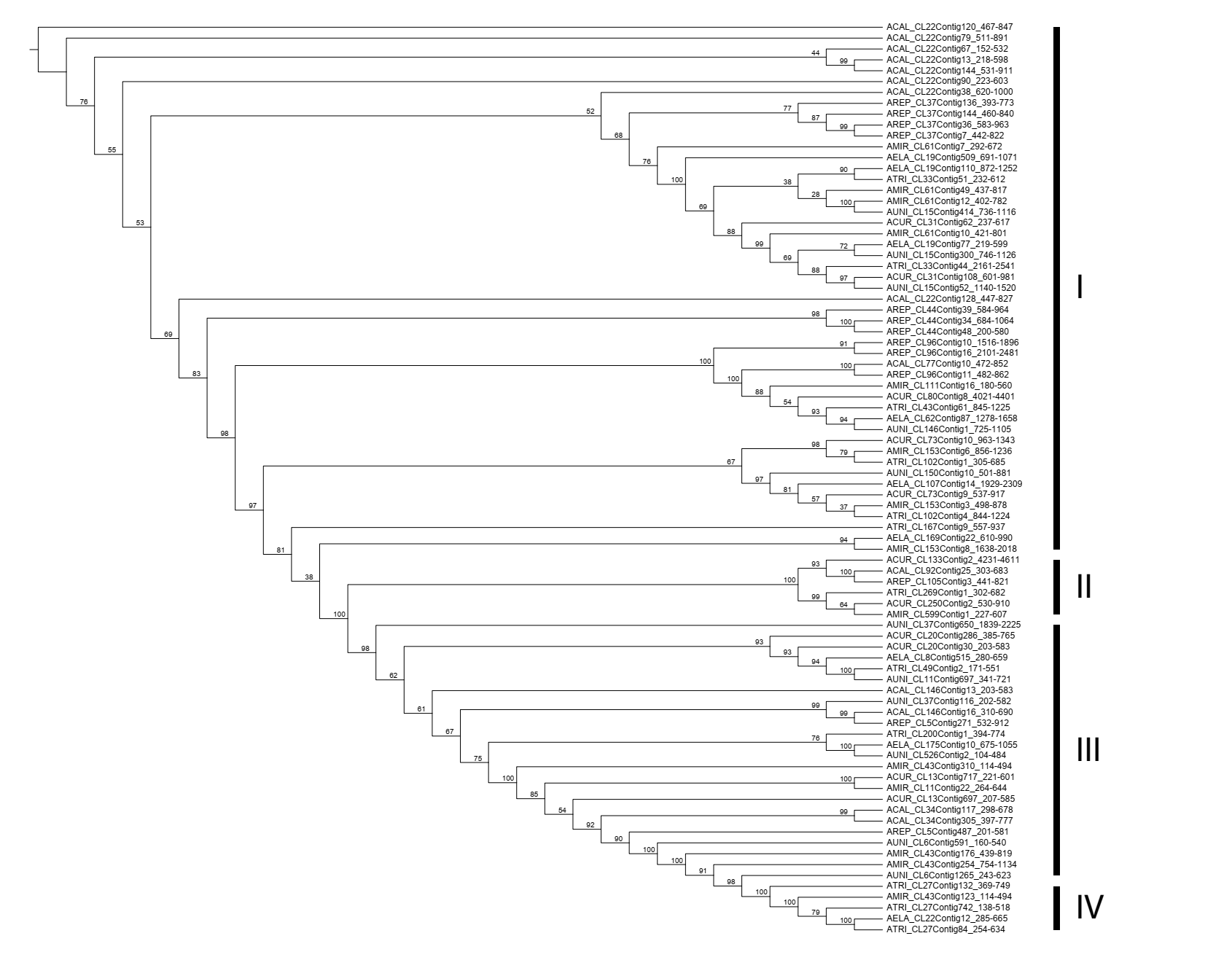


**Supplementary Figure 2.** Maximum likelihood phylogeny inferred from the GAG domain of the Tekay element across selected *Amomum* species.

**
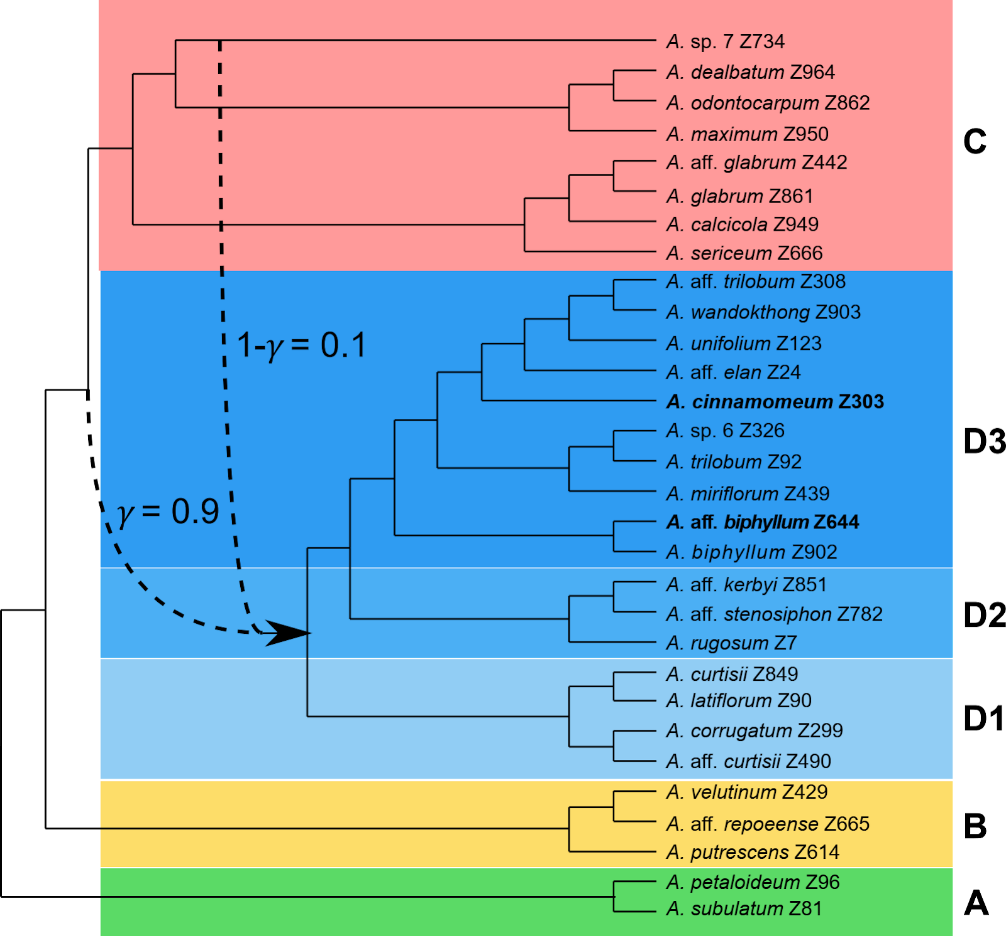
**

**Supplementary Figure 3.** PhyloNet network for 30 *Amomum* species. The best network resulting from the maximum pseudo-likelihood analysis of the '*complete dataset*' in Hyb-Seq (see Methods) represents the relationships among 30 species within the genus *Amomum*. Main clades (A, B, C, D) and subclades (D1, D2, D3) within the genus, as defined in Hlavatá et al. (2023), are distinguished by colors. Blue curves indicate the ancient hybrid origin of clade D. The 𝛾 and 1-𝛾 values denote the inheritance probability from the first and second possible ancestors, respectively. Tetraploids are indicated in bold.


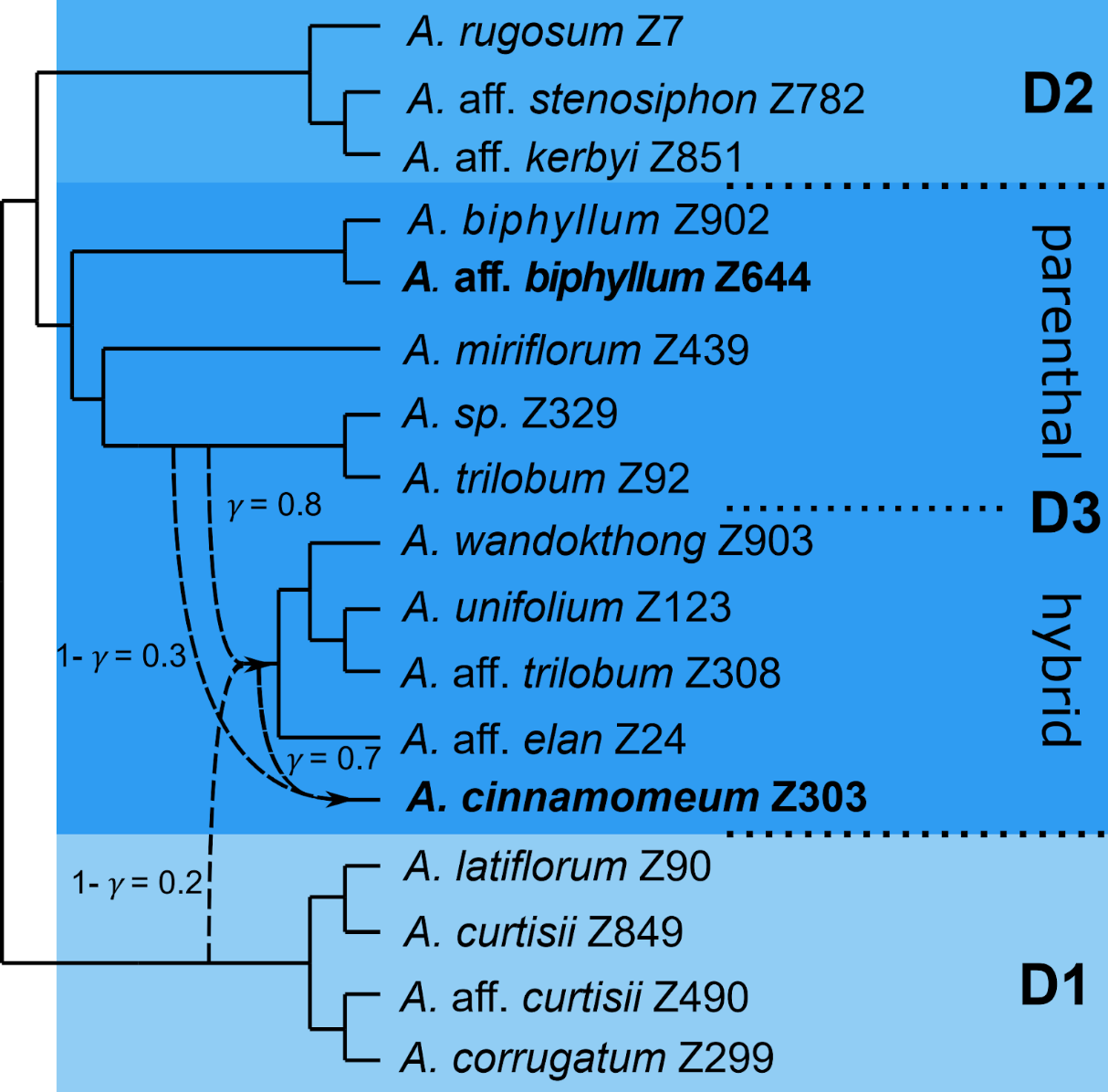


**Supplementary Figure 4.** PhyloNet network for *Amomum* clade D. The best network resulting from PhyloNet maximum pseudo-likelihood analysis of Hyb-Seq '*clade D dataset*' (see Methods), illustrating relationships within 17 *Amomum* species. Subclades within clade D (D1, D2, and D3) are distinguished by different shades of blue color. Tetraploids are highlighted in bold. *A.* = *Amomum*. Blue curves indicate the ancient hybrid origin of the 'D3 hybrid' subclade and the hybrid origin of tetraploid *A. cinnamomeum*. The 𝛾 and 1-𝛾 values denote the inheritance probability from the first and second possible ancestors, respectively.


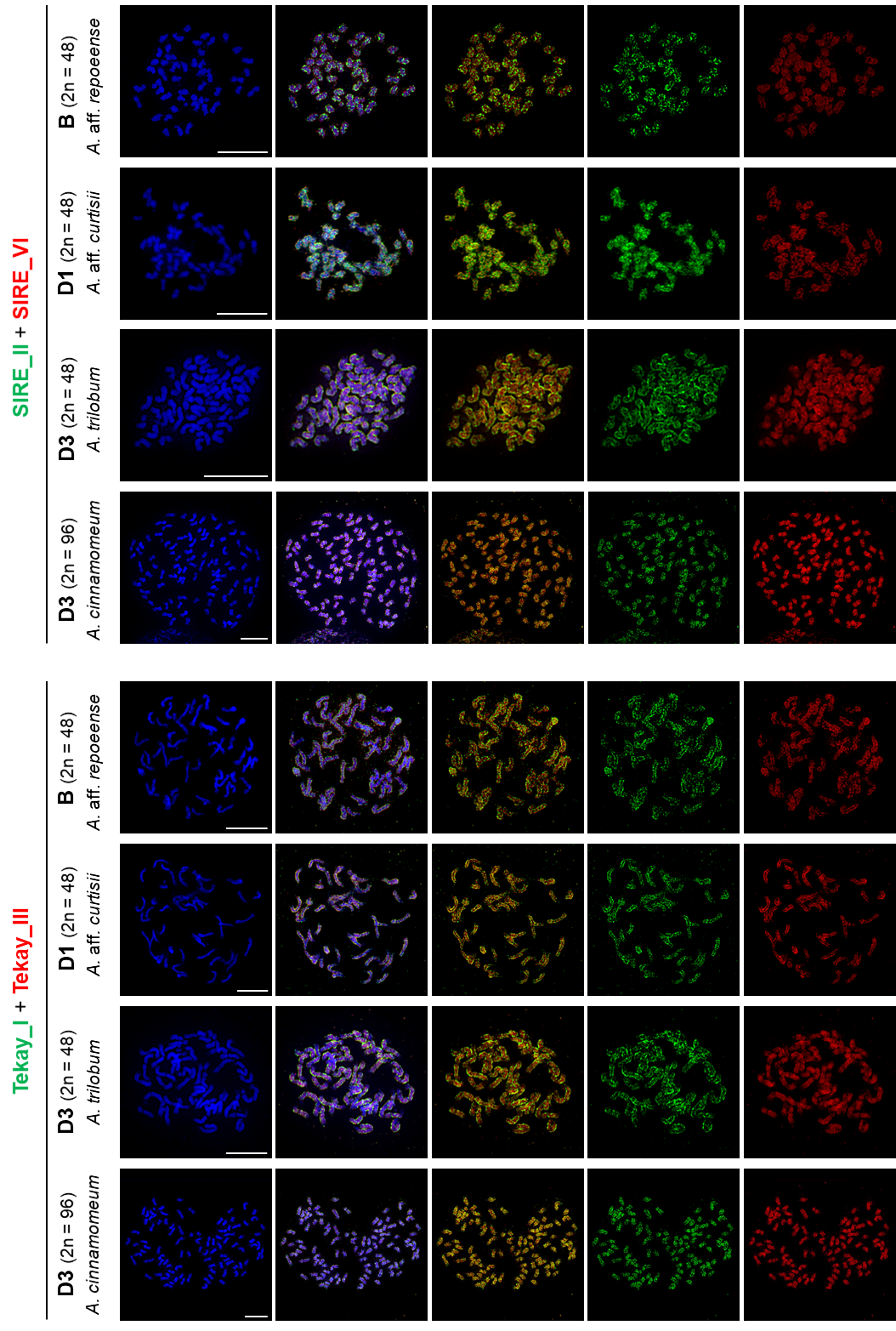


**Supplementary Figure 5.** FISH localization of the selected SIRE and Tekay elements on mitotic metaphase chromosomes of *Amomum* aff. *repoeense* (clade B, 2n = 48), *A.* aff. *curtisii* (clade D1, 2n = 48), *A. trilobum* (clade D3, 2n = 48), and *A. cinnamomeum* (clade D3, 2n = 96). Chromosomes were counterstained by DAPI; FISH signals are shown in colour as indicated. Scale bars, 10 µm.


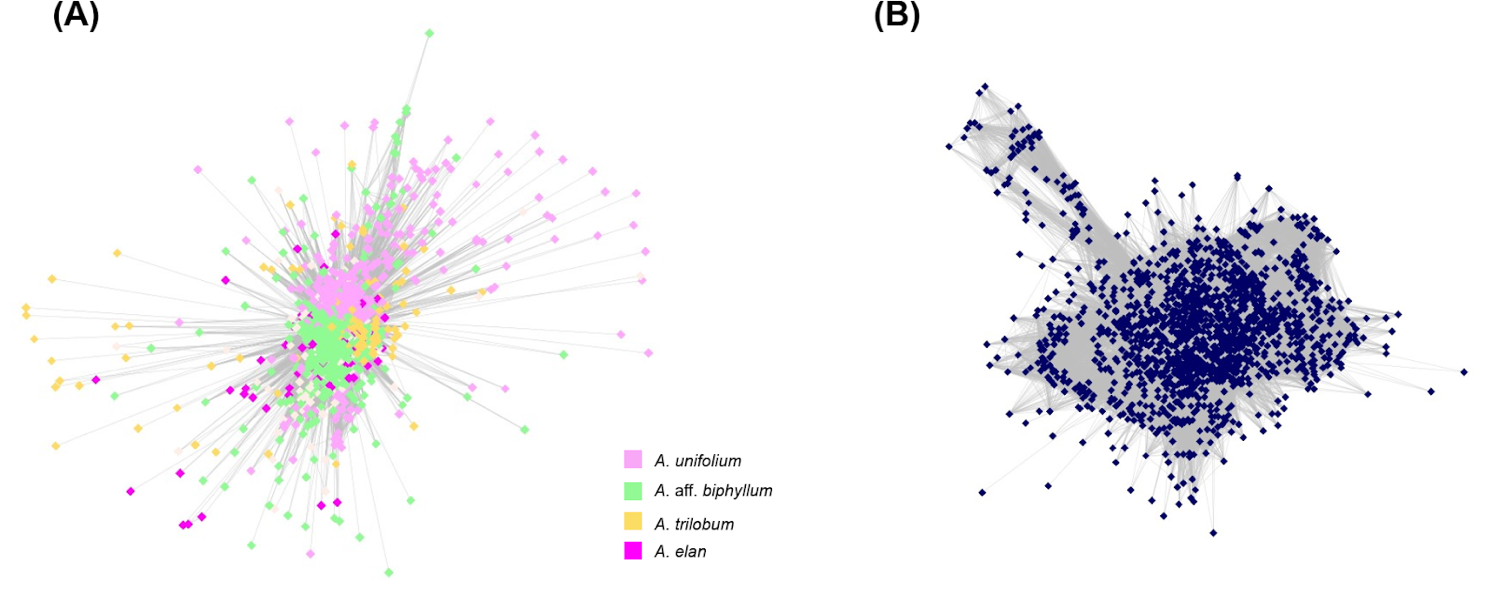


**Supplementary Figure 6.** Satellite cluster analysis in *Amomum* clades D3 and B. Satellite cluster CL48 is shared among multiple *Amomum* species within clade D3 (**A**), while satellite cluster CL185 is unique to *A. repoeense* within clade B (**B**). Adapted from RepeatExplorer. *A.* = *Amomum*.


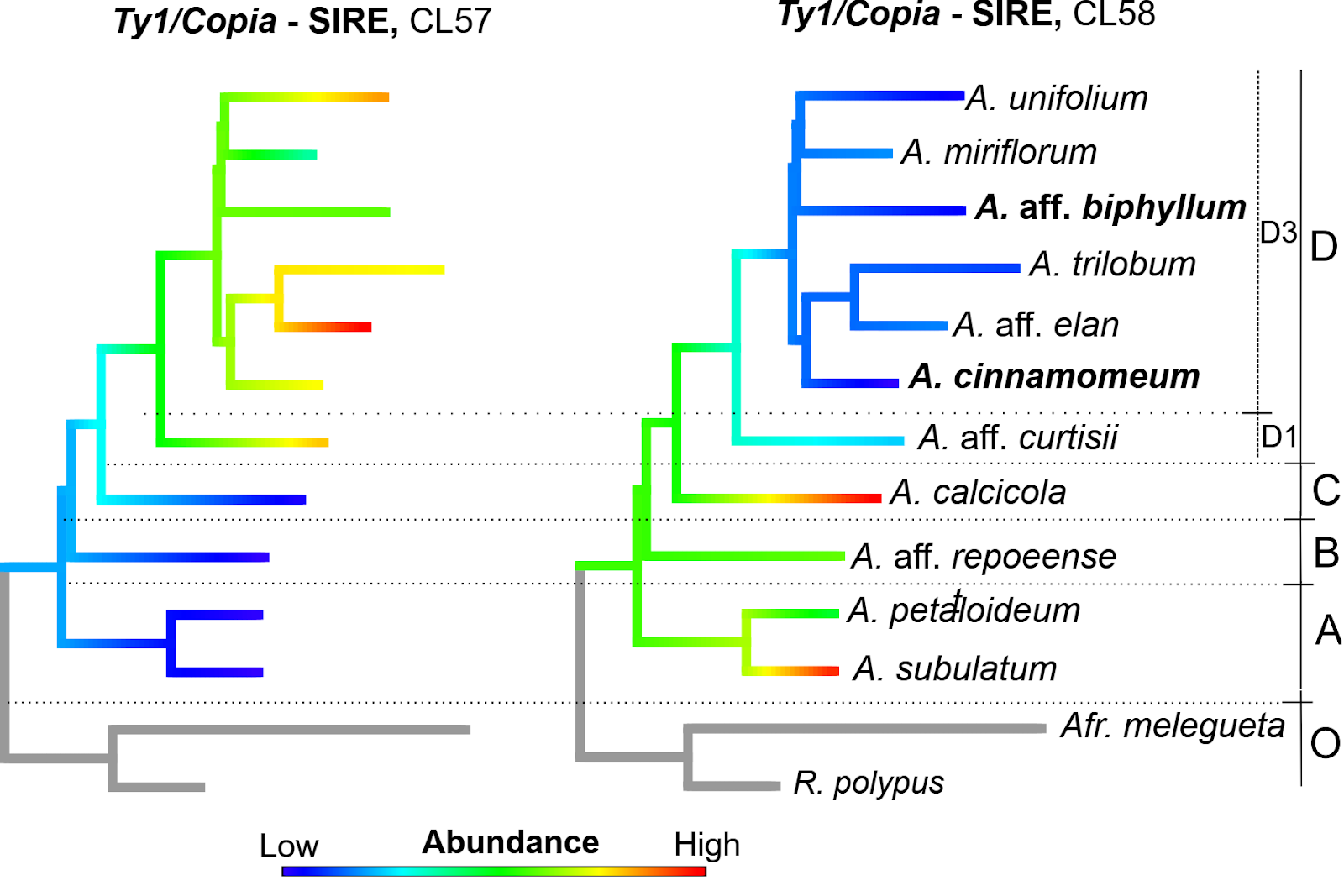


**Supplementary Figure 7.** Repeat sub-lineage abundances mapped onto *Amomum* phylogeny. Repeat sub-lineage (corresponding to clusters in comparative RepeatExplorer analysis) abundances are mapped onto the Hyb-Seq-based *Amomum* phylogeny. Two SIRE sub-lineages exhibit opposing trends (CL 57 amplification, CL 58 reduction), with abundances displaying a significant phylogenetic signal (Pagel’s λ = 1 and p < 0.05). Grey branches indicate missing data, and tetraploids are highlighted in bold. Phylogenetic clades are marked by lines and letters *Afr.* = *Aframomum.* *R.* = *Renealmia*. O = outgroup.

**
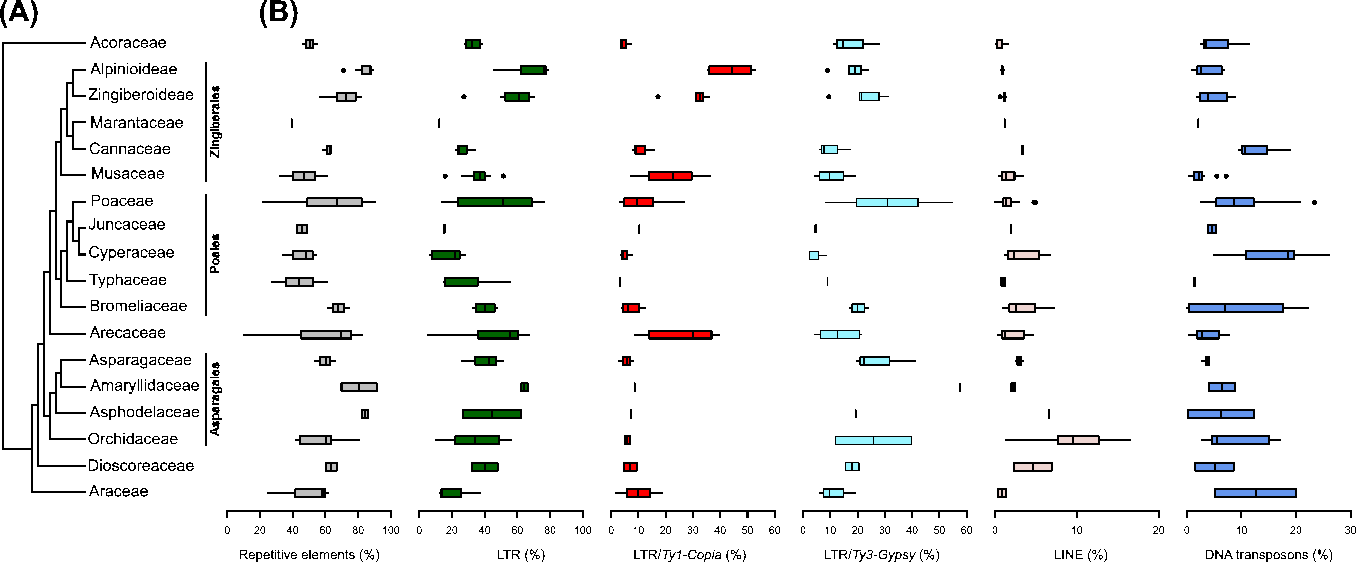
**

**Supplementary Figure 8.** Monocot plant family relationships and repeat abundances. (**A**) Phylogenetic relationships among 18 monocot plant families based on APG IV. (**B**) Percentages of overall repeat contents, along with the representation of LTRs, *Ty1-Copia*, *Ty3-Gypsy* superfamilies, LINEs, and DNA transposons in individual plant families, with data sources from selected genomic studies listed in Supplementary Table 4.

**3** **Tables**

**Supplementary Table 1.** List of investigated *Amomum* accessions and collection data. Accessions used in the study are listed, with herbarium specimen abbreviations in brackets where applicable. Herbarium abbreviations conform to the Index Herbariorum, except for NLS, which corresponds to the herbarium of Faculty of Sciences, National University of Laos as per Lamxay & Newman (2012). Collection details include: SBG - Singapore Botanic Gardens, SI - Smithsonian Institution, RBGE - Royal Botanic Gardens Edinburgh, PBG - Prague Botanic Garden. Abbreviations for different analyses are as follows: GS - 1C genome size visualisation in Figure 1, RE - RepeatExplorer analysis, PNc - PhyloNet *‘complete dataset’*, PND = PhyloNet *‘clade D dataset’*, SRR - number in Sequence Read Archive (SRA).

|  |  | **collection data** | | | **genomic characteristics** | | | **presence in analyses** | | | | **SRR code** | |
| --- | --- | --- | --- | --- | --- | --- | --- | --- | --- | --- | --- | --- | --- |
| **accession** | **clade** | **accession no.** | **herbarium specimen** | **country of origin** | **genome size (Mb/1C)** | **chromosome number (2n)** | **ploidy level** | **GS** | **RE** | **PNc** | **PND** | **HybSeq** | **genome skimming** |
| *Aframomum alboviolaceum* (Ridl.) K.Schum. | outgroup | Z236 | D.J. Harris 5745 (E) | Central African Rep. | **1213** |  | - | x | - | x | - | SRR7058219 | - |
| *Aframomum melegueta* K. Schum. | outgroup | Z743 | ex cult. RBGE 19982065 | Côte d'Ivoire | **1006** | - | - | x | x | x | - | SRR12824546 | SRR26415127 |
| *Amomum biphyllum* (Saensouk & P.Saensouk) Škorničk. & Hlavatá | D3 | Z902 | ex cult. Mahasarakham University | Thailand | **4699** | - | - | x | - | x | x | SRR12824540 | - |
| *Amomum biphyllum* aff. (Saensouk & P.Saensouk) Škorničk. & Hlavatá | D3 | Z644 | V. Lamxay VL2222 (NLS) | Laos | **6253** | 96 | 4x | x | x | x | x | SRR12824530 | SRR26415126 |
| *Amomum calcicola* Lamxay & M.F.Newman | C | Z949 | M. Newman et al. LAO 1302 (E) | Laos | **3115** | - | - | - | x | x | x | SRR12824535 | SRR26415122 |
| *Amomum cinnamomeum* Škorničk., Luu & H.Đ.Trần | D3 | Z303 | J. Leong-Škorničková GRC-393 (SING) | Vietnam | **7656** | 96 | 4x | x | x | x | x | SRR12824532 | SRR26415121 |
| *Amomum cinnamomeum* Škorničk., Luu & H.Đ.Trần | D3 | Z724 | ex cult. SI W.J. Kress & Q.J. Li 05-7779 | Vietnam | **7510** | - | 4x | x | - | - | - | - | - |
| *Amomum corrugatum* Škorničk., H.Đ.Trần & Luu | D1 | Z299 | H.Ɖ.Trấn et al. 53 (E, SING, VNM) | Vietnam | **2641** | 48 | 2x | x | - | x | x | SRR12824531 | - |
| *Amomum corrugatum* Škorničk., H.Đ.Trần & Luu | D1 | Z645 | H.Ɖ.Trấn et al. 52 (E, SING, VNM) | Vietnam | **2659** | 48 | 2x | x | - | - | - | - | - |
| Amomum curtisii (Baker) Škorničk. & Hlavatá | D1 | Z847 | ex cult. Singapore Botanic Gardens | Pen. Malaysia | 2975 | - | - | x | - | - | - | SRR12824539 | SRR26415120 |
| *Amomum curtisii* aff. (Baker) Škorničk. & Hlavatá | D1 | Z490 | ex cult. RBGE 20001425 | Thailand | **2782** | 48 | 2x | x | - | x | x | SRR12824538 | - |
| *Amomum curtisii* aff. (Baker) Škorničk. & Hlavatá | D1 | Z721 | W.J. Kress 99-6322 | Thailand | **2771** | - | - | x | - | - | - | - | - |
| *Amomum curtisii* aff. (Baker) Škorničk. & Hlavatá | D1 | Z739 | J. Mood 13P14 (PRC) | Malaysia | **2906** | - |  | x | - | - | - | - | - |
| *Amomum dealbatum* Roxb. | C | Z964 | ex cult. SI W.J. Kress 06-8414 | China | **3110** | - | - | x | - | x | x | SRR12824524 | - |
| *Amomum elan* aff. (C.K. Lim) Škorničk. & Hlavatá | D3 | Z024 | J. Leong-Škorničková GRC-079 (SING) | Malaysia | **3863** | - | - | x | x | x | x | SRR12824528 | SRR26415119 |
| *Amomum elan* aff. (C.K. Lim) Škorničk. & Hlavatá | D3 | Z732 | J. Mood 1286 (PRC) | Malaysia, Sabah | **3585** | - | - | x | - | - | - | - | - |
| *Amomum glabrum* aff. S.Q. Tong | C | Z442 | J. Leong-Škorničková et al. JLS-1598 (E, PR, SING, VNMN) | Vietnam | **3115** | - | - | x | - | x | x | SRR12824517 | - |
| *Amomum glabrum* S.Q. Tong | C | Z861 | V. Lamxay VL1157 (E, NLS) | Laos | **3110** | - | - | x | - | x | x | SRR12824516 | - |
| *Amomum kerbyi* aff. (R.M. Sm.) Škorničk. & Hlavatá | D2 | Z851 | ex cult. PBG M. Dančák 2015/3004 | Brunei | **3105** | - | - | x | - | x | x | SRR12824537 | - |
| *Amomum latiflorum* (Ridl.) Škorničk. & Hlavatá | D1 | Z090 | J. Leong-Škorničková et al. SNG-13 (SING) | Singapore | **3139** | - | - | x | - | x | x | SRR12824536 | - |
| *Amomum latiflorum* (Ridl.) Škorničk. & Hlavatá | D1 | Z121 | W.J. Kress 99-6322 | Thailand | **3156** | - | - | x | - | - | - | - | - |
| *Amomum latiflorum* (Ridl.) Škorničk. & Hlavatá | D1 | Z642 | H. Ibrahim & S. Teo SNG-113 (SING) | Singapore | **3086** | - | - | x | - | - | - | - | - |
| *Amomum maximum* Roxb. | C | Z950 | A.D. Poulsen 2920 (E) | Papua New Guinea | **2748** | - | - | x | - | x | x | SRR12824515 | - |
| *Amomum maximum* Roxb. | C | Z686 | Leong-Škorničková et al. JLS-1726 (SING, PR, P, E) | Laos | **2749** | - | - | x | - | - | - | - | - |
| *Amomum miriflorum* Škorničk. & Q.B.Nguyen | D3 | Z439 | J. Leong-Škorničková et al. JLS-1589 (E, K, P, PR, SING, VNMN) | Vietnam | **4294** | 48 | 2x | x | x | x | x | SRR12824534 | - |
| *Amomum odontocarpum* D. Fang | C | Z862 | V. Lamxay VL1300 (E, NLS) | Laos | **-** | - | - | - | - | x | x | SRR12824514 | - |
| *Amomum petaloideum* (S.Q.Tong) T.L.Wu | A | Z096 | W.J. Kress et al. 95-5508 (US) | China | **3078** | - | - | x | x | x | x | SRR12824513 | SRR26415117 |
| *Amomum putrescens* D. Fang | B | Z614 | J. Leong-Škorničková et al. JLS-2146 (VNM, SING) | Vietnam | **2592** | - | - | x | - | x | x | SRR12824545 | - |
| *Amomum ranongense (Picheans. & Yupparach) Škorničk. & Hlavatá* | D2 | Z731 | J. Mood 3377 (PRC) | Thailand | **3016** | - | - | x | - | - | - | - | - |
| *Amomum ranongense* aff. *(Picheans. & Yupparach) Škorničk. & Hlavatá* | D2 | Z727 | J. Mood 08P288 (PRC) | Thailand | **2914** | - | - | x | - | - | - | - | - |
| *Amomum repoeense* aff. Pierre ex Gagnep. | B | Z665 | H.Ɖ. Trấn et al. 67 (E, SING, VNM) | Vietnam | **2465** | 48 | 2x | x | x | x | x | SRR12824544 | SRR26415116 |
| *Amomum repoeense* aff. Pierre ex Gagnep. | B | Z450 | J. Leong-Škorničková  JLS-1619 (SING, E, P, VNMN) | Vietnam | **2761** | - | - | x | - | - | - | - | - |
| *Amomum repoeense* aff. Pierre ex Gagnep. | B | Z456 | J. Leong-Škorničková JLS-1637 (SING, E, P, VNMN) | Vietnam | **2578** | - | -- | x | - | - | - | - | - |
| *Amomum repoeense* aff. Pierre ex Gagnep. | B | Z659 | V. Lamxay VL2223 RBGE20111045 | Laos | **2344** | - | - | x | - | - | - | - | - |
| *Amomum rugosum* (Y.K.Kam) Škorničk. & Hlavatá | D2 | Z007 | J. Leong-Škorničková GRC-363 (SING) | Malaysia | **2910** | - | - | x | - | x | x | SRR12824533 | - |
| *Amomum sericeum* Roxb. | C | Z662 | M.F. Newman 2397 (E) | Cambodia | **3174** | - | - | x | - | x | x | SRR12824543 | - |
| *Amomum sericeum* Roxb. | C | Z559 | J. Leong-Škorničková et al. JLS-1675 (E, SING) | Laos | **3646** | - | - | x | - | - | - | - | - |
| *Amomum smithiae* (Y.K.Kam) Škorničk. & Hlavatá | D2 | Z720 | ex cult. SBG20001092 | Malaysia | **3179** | - | - | x | - | - | - | - | - |
| *Amomum sp. 6* | D3 | Z329 | ex cult. SBG20110992 | Vietnam | **3130** | 48 | 2x | x | - | x | x | SRR12824527 | - |
| *Amomum sp. 7* | C | Z734 | J. Mood 3387 (PRC) | Thailand | **2339** | - | - | x | - | x | - | SRR12824542 | - |
| *Amomum sp. 8* | C | Z640 | ex cult. SBG20122011 | Laos | **2389** | - | - | x | - | - | - | - | - |
| *Amomum sp. 9* | B | Z491 | H.Ɖ.Trấn 366 (VNM) | Vietnam | **2337** | - | - | x | - | - | - | - | - |
| *Amomum stenosiphon* aff. K.Schum. | D2 | Z872 | Conlon et al. 41 (E) | Indonesia | **3037** | - | - | x | - | x | x | SRR12824526 | - |
| *Amomum stenosiphon* aff. K.Schum. | D2 | Z725 | J. Mood 89P43 (PRC) | Malaysia, Sabah | **3038** | - | - | x | - | - | - | - | - |
| *Amomum subulatum* Roxb. | A | Z081 | J. Škorničková CU71468 (CALI, SING) | India | **1731** | 48 | 2x | x | x | x | x | SRR12824541 | SRR26415115 |
| *Amomum trilobum* aff. Gagnep. | D3 | Z092 | M.F. Newman & J. Škorničková 1455 (E, SING) | Vietnam | **3750** | 48 | 2x | x | x | x | x | SRR12824525 | SRR26415125 |
| *Amomum trilobum* aff. Gagnep. | D3 | Z308 | ex cult. SBG 20110993 | Vietnam | **3760** | 48 | 2x | x | - | x | x | SRR12824529 | - |
| *Amomum unifolium* aff. Gagnep. | D3 | Z304 | ex cult. SBG20110994 | Vietnam | **2958** | - | - | x | - | - | - | - | - |
| *Amomum unifolium* aff. Gagnep. | D3 | Z636 | ex cult. SBG20123278 | Vietnam | **2900** | - | - | x | - | - | - | - | - |
| *Amomum unifolium* aff. Gagnep. | D3 | Z368 | H.Ɖ. Trấn 257 (VNM) | Vietnam | **2890** | - | - | x | - | - | - | - | - |
| *Amomum unifolium* aff. Gagnep. | D3 | Z661 | H.Ɖ. Trấn s.n. RBGE20081123 | Vietnam | **2993** | - | - | x | - | - | - | - | - |
| *Amomum unifolium* aff. Gagnep. | D3 | Z850 | ex cult. SBG20111709 | Vietnam | **3120** | - | - | x | - | - | - | - | - |
| *Amomum unifolium* Gagnep. | D3 | Z123 | M.F. Newman & J. Škorničková 2002 (E, SING) | Vietnam | **2927** | 48 | 2x | x | x | x | x | SRR12824523 | SRR26415124 |
| *Amomum velutinum* X.E.Ye, Škorničk. & N.H.Xia | B | Z429 | J. Leong-Škorničková et al. JLS-1557 (E, PR, SING, VNMN) | Vietnam | **2557** | - | - | x | - | x | x | SRR12824512 | - |
| *Amomum wandokthong* (Picheans. & Yupparach) Škorničk. & Hlavatá | D3 | Z903 | K. Hlavata 1 (PRC) | Thailand | **3902** | - | - | x | - | x | x | SRR12824522 | - |
| *Amomum wandokthong* (Picheans. & Yupparach) Škorničk. & Hlavatá | D3 | Z730 | J. Mood 3000 (PRC) | Thailand | **7679** | - | - | x | - | - | - | - | - |
| *Renealmia polypus* Gagnep. | outgroup | Z575 | M. Newman & J. Škorničková 2009 (E) | Central African Rep. | **1224** | - | - | - | x | x | - | SRR7058219 | SRR26415123 |

**Supplementary Table 2.** Repeat percentages of individual species calculated in the RepeatExplorer analysis. Subtotals that are part of the final repeatome percentage are highlighted in bold. Subtotals of *Ty1-Copia* and *Ty3-Gypsy* superfamilies are bolded and italicized. O = outgroup. *R.* = *Renealmia*. *Afr.* = *Aframomum*. *A*. = *Amomum*.

| **Species** | | | ***R. polypus*** | ***Afr. melegueta*** | ***A. subulatum*** | ***A.* *petaloideum*** | ***A.* aff. *repoeense*** | ***A. calcicola*** | ***A.* aff. *curtisii*** | ***A. cinnamomeum*** | ***A.* aff. *elan*** | ***A. trilobum*** | ***A. aff. biphyllum*** | ***A. miriflorum*** | ***A. unifolium*** |  |
| --- | --- | --- | --- | --- | --- | --- | --- | --- | --- | --- | --- | --- | --- | --- | --- | --- |
| **Clade** | | | **O** | **O** | **A** | **A** | **B** | **C** | **D1** | **D3** | **D3** | **D3** | **D3** | **D3** | **D3** |  |
| **Chromosome number (2n)** | | | **NA** | **NA** | **48** | **NA** | **48** | **NA** | **48** | **96** | **48** | **48** | **96** | **48** | **48** |  |
| **Repeat group** | | **Lineage** | **Proportion (%)** | | | | | | | | | | | | | |
| ***Ty1-Copia* – total** | | | ***19.84*** | ***23.72*** | ***40.33*** | ***48.27*** | ***45.93*** | ***50.80*** | ***43.46*** | ***29.15*** | ***44.11*** | ***37.72*** | ***41.74*** | ***31.82*** | ***31.65*** |  |
|  | *Ty1-Copia* unclassified | | 0.11 | 6.79 | 11.60 | 3.03 | 13.85 | 1.24 | 7.16 | 0.00 | 0.17 | 0.00 | 0.64 | 14.01 | 4.10 |  |
|  | Alesia |  | 0.00 | 0.22 | 0.00 | 0.00 | 0.00 | 0.00 | 0.00 | 0.00 | 0.00 | 0.00 | 0.00 | 0.00 | 0.00 |  |
|  | Ale |  | 0.67 | 0.00 | 0.05 | 0.07 | 0.00 | 0.11 | 0.63 | 0.77 | 0.58 | 0.65 | 0.80 | 0.42 | 0.69 |  |
|  | Angela |  | 17.29 | 12.26 | 14.65 | 18.77 | 19.90 | 17.61 | 9.68 | 4.53 | 9.98 | 9.46 | 6.90 | 5.99 | 6.79 |  |
|  | Ikeros |  | 0.69 | 0.82 | 0.59 | 0.43 | 0.42 | 0.35 | 0.40 | 0.54 | 0.43 | 0.40 | 0.43 | 0.32 | 0.43 |  |
|  | Ivana |  | 0.47 | 0.39 | 0.02 | 0.02 | 0.00 | 0.00 | 0.03 | 0.04 | 0.06 | 0.07 | 0.06 | 0.02 | 0.09 |  |
|  | SIRE |  | 0.08 | 0.04 | 13.16 | 25.67 | 11.57 | 31.24 | 25.19 | 22.66 | 32.42 | 26.79 | 32.44 | 10.77 | 19.14 |  |
|  | TAR |  | 0.40 | 0.78 | 0.27 | 0.28 | 0.19 | 0.27 | 0.35 | 0.58 | 0.45 | 0.32 | 0.45 | 0.29 | 0.40 |  |
|  | Tork |  | 0.13 | 2.42 | 0.00 | 0.00 | 0.00 | 0.00 | 0.01 | 0.03 | 0.02 | 0.03 | 0.02 | 0.00 | 0.01 |  |
| ***Ty3-Gypsy* – total** | | | ***22.43*** | ***12.13*** | ***25.15*** | ***13.41*** | ***19.70*** | ***19.23*** | ***15.08*** | ***20.59*** | ***21.17*** | ***23.26*** | ***17.3*** | ***19.54*** | ***21.14*** |  |
|  | *Ty3-Gypsy* unclassified | | 1.58 | 0.81 | 1.28 | 1.15 | 1.26 | 0.26 | 1.64 | 1.67 | 0.36 | 0.78 | 0.85 | 2.28 | 5.15 |  |
|  | Chromovirus unclassified | | 0.00 | 0.00 | 0.00 | 0.00 | 0.00 | 0.00 | 0.00 | 0.00 | 0.00 | 0.00 | 0.01 | 0.00 | 0.00 |  |
|  | CRM |  | 1.19 | 1.22 | 0.12 | 0.18 | 0.15 | 0.27 | 0.39 | 0.21 | 0.48 | 0.54 | 0.27 | 0.12 | 0.20 |  |
|  | Galadriel |  | 0.00 | 0.00 | 0.00 | 0.01 | 0.00 | 0.00 | 0.00 | 0.00 | 0.00 | 0.00 | 0.00 | 0.00 | 0.00 |  |
|  | Tekay |  | 1.29 | 0.88 | 17.47 | 7.17 | 10.98 | 13.10 | 9.75 | 15.25 | 15.08 | 16.71 | 11.93 | 12.97 | 12.60 |  |
|  | OTA unclassified | | 0.00 | 0.00 | 0.00 | 0.00 | 0.00 | 0.00 | 0.00 | 0.00 | 0.00 | 0.00 | 0.00 | 0.00 | 0.00 |  |
|  | Athila |  | 18.11 | 0.69 | 2.67 | 1.89 | 3.15 | 2.31 | 1.49 | 1.20 | 3.73 | 3.92 | 1.12 | 1.86 | 1.05 |  |
|  | Retand |  | 0.26 | 8.52 | 3.61 | 3.02 | 4.16 | 3.29 | 1.81 | 2.25 | 1.53 | 1.31 | 3.14 | 2.31 | 2.13 |  |
| LTR unclassifed | | | 5.61 | 2.33 | 2.01 | 1.98 | 2.25 | 2.56 | 1.50 | 10.29 | 0.53 | 2.40 | 1.53 | 7.24 | 4.66 |  |
| **LTR – total** | | | **47.87** | **38.16** | **67.50** | **63.66** | **67.89** | **72.60** | **60.03** | **60.04** | **65.80** | **63.38** | **60.58** | **58.59** | **57.45** |  |
| **LINE** | | | **0.43** | **2.48** | **0.81** | **0.59** | **0.65** | **1.01** | **0.67** | **3.73** | **0.92** | **0.88** | **1.10** | **1.23** | **0.85** |  |
| **Pararetrovirus** **and Class I unclassified** | | | **0.32** | **0.10** | **0.00** | **0.02** | **0.00** | **0.02** | **0.01** | **1.32** | **0.02** | **0.87** | **0.00** | **0.00** | **0.00** |  |
| **DNA transposons – total** | | | **0.46** | **0.45** | **0.64** | **0.51** | **0.27** | **0.38** | **0.56** | **0.59** | **1.43** | **0.47** | **0.44** | **0.27** | **0.61** |  |
|  | DNA transp. unclassified | | 0.00 | 0.00 | 0.03 | 0.00 | 0.00 | 0.00 | 0.10 | 0.00 | 0.00 | 0.00 | 0.00 | 0.00 | 0.00 |  |
|  | EnSpm_CACTA | | 0.09 | 0.36 | 0.16 | 0.26 | 0.16 | 0.16 | 0.23 | 0.21 | 0.38 | 0.17 | 0.18 | 0.17 | 0.28 |  |
|  | hAT | | 0.36 | 0.09 | 0.00 | 0.00 | 0.00 | 0.00 | 0.04 | 0.10 | 0.06 | 0.05 | 0.00 | 0.00 | 0.05 |  |
|  | Mudr_Mutator | | 0.00 | 0.00 | 0.45 | 0.25 | 0.10 | 0.22 | 0.19 | 0.28 | 0.99 | 0.24 | 0.25 | 0.09 | 0.28 |  |
| **rDNA – total** | | | **2.01** | **6.00** | **0.78** | **0.46** | **0.73** | **1.91** | **2.34** | **1.37** | **0.86** | **2.39** | **1.70** | **1.20** | **5.06** |  |
|  | rDNA unclassified | | 0.80 | 0.00 | 0.07 | 0.00 | 0.00 | 0.00 | 0.00 | 0.00 | 0.00 | 0.00 | 0.00 | 0.00 | 0.04 |  |
|  | 45S_rDNA | | 0.00 | 5.88 | 0.71 | 0.45 | 0.73 | 1.89 | 2.30 | 1.35 | 0.84 | 2.37 | 1.68 | 1.18 | 4.99 |  |
|  | 18S_rDNA | | 1.16 | 0.00 | 0.00 | 0.00 | 0.00 | 0.00 | 0.02 | 0.00 | 0.00 | 0.00 | 0.00 | 0.00 | 0.00 |  |
|  | 5S_rDNA | | 0.05 | 0.12 | 0.00 | 0.01 | 0.00 | 0.02 | 0.02 | 0.02 | 0.02 | 0.01 | 0.02 | 0.02 | 0.03 |  |
| **Satellite** | | | **0.00** | **0.06** | **1.72** | **1.52** | **1.39** | **1.38** | **4.14** | **2.36** | **1.66** | **1.16** | **3.38** | **2.95** | **0.00** |  |
| **Small clusters** | | | **22.46** | **26.06** | **13.43** | **16.54** | **13.49** | **9.85** | **16.15** | **14.20** | **14.36** | **13.90** | **16.55** | **16.86** | **16.05** |  |
| **Other unclassified** | | | **1.88** | **2.64** | **3.40** | **3.69** | **2.68** | **0.52** | **0.98** | **3.85** | **2.06** | **2.33** | **2.95** | **3.55** | **4.84** |  |
| **Single copy** **sequences** | | | 24.60 | 24.07 | 11.72 | 13.02 | 12.91 | 12.33 | 15.11 | 12.56 | 12.89 | 14.63 | 13.30 | 15.36 | 15.13 |  |
| **1C genome size (Mbp)** | | | 1224 | 1006 | 1731 | 3078 | 2465 | 3081 | 2783 | 7656 | 3863 | 3750 | 6254 | 4294 | 2927 |  |
| **Total repeats (%)** | | | **75.43** | **75.96** | **88.27** | **86.99** | **87.08** | **88.64** | **84.89** | **87.44** | **87.12** | **85.37** | **86.70** | **84.65** | **84.87** |  |

**Supplementary Table 3.** Correlation between individual repeat groups, superfamilies, and lineages with GS and their phylogenetic signal (Pagel's lambda). Significant values are denoted in bold.

| **Repeats** | | | | **Correlation with GS** | | **Phylogenetic signal** | |
| --- | --- | --- | --- | --- | --- | --- | --- |
| **Group** | **Superfamily** | Family | **Lineage** | **Adj. R2** | **p-value** | **λ** | **p-value** |
| LTR-retrotransposons | *Ty1-Copia* | | Total *Ty1-Copia* | **0.23** | **0.06** | 0.79 | 0.08 |
|  |  |  | Ale | 0.55 | 0.002 | **1.00** | **<0.001** |
|  |  |  | Angela | -0.08 | 0.76 | 0.42 | 0.49 |
|  |  |  | Ikeros | **0.77** | **<0.001** | 0.35 | 0.53 |
|  |  |  | Ivana | -0.05 | 0.50 | **1.00** | **0.05** |
|  |  |  | SIRE | **0.40** | **0.01** | 0.08 | 0.90 |
|  |  |  | TAR | **0.76** | **<0.001** | 0.77 | 0.10 |
|  |  |  | Tork | -0.06 | 0.62 | 0.49 | 0.29 |
|  | *Ty3-Gypsy* |  | Total *Ty3-Gypsy* | **0.23** | **0.06** | **1.00** | **0.01** |
|  |  | Chromovirus | CRM | -0.08 | 0.70 | 0.19 | 0.69 |
|  |  | Chromovirus | Tekay | **0.48** | **0.004** | 0.96 | 0.09 |
|  |  | Non-chromovirus | Athila | -0.01 | 0.38 | 0.00 | 1.00 |
|  |  | Non-chromovirus | Retand | 0.10 | 0.14 | 0.00 | 1.00 |
| LINE | | | | **0.66** | **<0.001** | 0.00 | 1.00 |
| Unclassified LTR | | | | -0.08 | 0.76 | 0.00 | 1.00 |
| Pararetrovirus and Class I unclassified | | | | **0.33** | **0.02** | 0.00 | 1.00 |
| DNA transposons | | | Total DNA transposons | 0.03 | 0.44 | 0.11 | 0.84 |
|  |  |  | EnSpm/CACTA | 0.21 | 0.07 | 0.15 | 0.81 |
|  |  |  | hAT | -0.04 | 0.50 | 0.22 | 0.66 |
|  |  |  | MuDR-Mutator | -0.06 | 0.62 | 0.00 | 1.00 |
| Other unclassified | | | | **0.35** | **0.02** | 0.09 | 0.93 |
| Satellites | | | | **0.42** | **0.01** | 0.65 | 0.23 |
| rDNA | | | | -0.08 | 0.74 | 0.36 | 0.48 |
| Total repeats | | | | **0.40** | **0.01** | **1.00** | **0.01** |

**Supplementary Table 4.** Repeat content in monocot families. Data sourced for repeat abundances in monocot (sub)families from selected genomic studies up to September 2023, accessible at <https://www.plabipd.de>, with references and links provided.

| **(sub)family** | **species** | **repetitive sequences (%)** | **LTRs (%)** | **Ty1-Copia (%)** | **Ty3-Gypsy (%)** | **LINE (%)** | **DNA transposons (%)** | **reference** | **download link** |
| --- | --- | --- | --- | --- | --- | --- | --- | --- | --- |
| Acoraceae | *Acorus calamus* | 46.30 | 28.50 | 3.57 | 11.27 | 0.24 | 3.55 | Ma, L., Liu, K.W., Li, Z., Hsiao, Y.Y., Qi, Y., Fu, T., Tang, G.D., Zhang, D., Sun, W.H., Liu, D.K. and Li, Y. 2023. Diploid and tetraploid genomes of Acorus and the evolution of monocots. Nature Communications, 14(1), p.3661. | https://www.nature.com/articles/s41467-023-38829-3 |
| Acoraceae | *Acorus calamus* | 54.82 | 28.20 | 3.77 | 13.64 | NA | 11.46 | Shi, T., Huneau, C., Zhang, Y., Li, Y., Chen, J., Salse, J. and Wang, Q. 2022. The slow-evolving Acorus tatarinowii genome sheds light on ancestral monocot evolution. Nature Plants, 8(7), pp.764-777. | https://www.nature.com/articles/s41477-022-01187-x |
| Acoraceae | *Acorus gramineus* | 50.07 | 38.40 | 7.20 | 28.00 | 1.59 | NA | Guo, X., Wang, F., Fang, D., Lin, Q., Sahu, S.K., Luo, L., Li, J., Chen, Y., Dong, S., Chen, S. and Liu, Y. 2023. The genome of Acorus deciphers insights into early monocot evolution. Nature Communications, 14(1), p.3662. | https://www.nature.com/articles/s41467-023-38836-4#Sec2 |
| Acoraceae | *Acorus gramineus* | 50.56 | 35.61 | 3.91 | 15.93 | 0.17 | 2.57 | Ma, L., Liu, K.W., Li, Z., Hsiao, Y.Y., Qi, Y., Fu, T., Tang, G.D., Zhang, D., Sun, W.H., Liu, D.K. and Li, Y. 2023. Diploid and tetraploid genomes of Acorus and the evolution of monocots. Nature Communications, 14(1), p.3661. | https://www.nature.com/articles/s41467-023-38829-3 |
| Amaryllidaceae | *Allium fistulosum* | 69.81 | 62.18 | NA | NA | 2.00 | 4.00 | Liao, N., Hu, Z., Miao, J., Hu, X., Lyu, X., Fang, H., Zhou, Y.M., Mahmoud, A., Deng, G., Meng, Y.Q. and Zhang, K. 2022. Chromosome-level genome assembly of bunching onion illuminates genome evolution and flavor formation in Allium crops. Nature Communications, 13(1), p.6690. | http://dx.doi.org/10.1038/s41467-022-34491-3 |
| Amaryllidaceae | *Allium sativum* | 91.30 | 66.20 | 8.60 | 57.50 | 2.50 | 8.90 | Sun, X., Zhu, S., Li, N., Cheng, Y., Zhao, J., Qiao, X., Lu, L., Liu, S., Wang, Y., Liu, C. and Li, B. 2020. A chromosome-level genome assembly of garlic (Allium sativum) provides insights into genome evolution and allicin biosynthesis. Molecular Plant, 13(9), pp.1328-1339. | http://dx.doi.org/10.1016/j.molp.2020.07.019 |
| Araceae | *Lemna minor* | 61.50 | NA | 18.79 | 10.59 | 1.35 | 5.08 | Van Hoeck, A., Horemans, N., Monsieurs, P., Cao, H.X., Vandenhove, H. and Blust, R. 2015. The first draft genome of the aquatic model plant Lemna minor opens the route for future stress physiology research and biotechnological applications. Biotechnology for Biofuels, 8(1), pp.1-13. | http://dx.doi.org/10.1186/s13068-015-0381-1 |
| Araceae | *Lemna minuta* | 58.20 | 37.40 | 9.80 | 19.30 | NA | 20.07 | Abramson, B.W., Novotny, M., Hartwick, N.T., Colt, K., Aevermann, B.D., Scheuermann, R.H. and Michael, T.P. 2022. The genome and preliminary single-nuclei transcriptome of Lemna minuta reveals mechanisms of invasiveness. Plant Physiology, 188(2), pp.879-897. | http://dx.doi.org/10.1093/plphys/kiab564 |
| Araceae | *Spirodela intermedia* | 25.00 | 14.10 | NA | 8.96 | 0.37 | NA | Hoang, P.T., Fiebig, A., Novák, P., Macas, J., Cao, H.X., Stepanenko, A., Chen, G., Borisjuk, N., Scholz, U. and Schubert, I. 2020. Chromosome-scale genome assembly for the duckweed Spirodela intermedia, integrating cytogenetic maps, PacBio and Oxford Nanopore libraries. Scientific reports, 10(1), p.19230. | https://www.nature.com/articles/s41598-020-75728-9 |
| Araceae | *Spirodela polyrhiza* | NA | 13.00 | 1.72 | 6.06 | NA | NA | Wang, W., Haberer, G., Gundlach, H., Gläßer, C., Nussbaumer, T.C.L.M., Luo, M.C., Lomsadze, A., Borodovsky, M., Kerstetter, R.A., Shanklin, J. and Byrant, D.W. 2014. The Spirodela polyrhiza genome reveals insights into its neotenous reduction fast growth and aquatic lifestyle. Nature communications, 5(1), p.3311. | https://www.nature.com/articles/ncomms4311 |
| Arecaceae | *Areca catechu* | 82.20 | 55.20 | NA | NA | 3.58 | 5.80 | Yang, Y., Huang, L., Xu, C., Qi, L., Wu, Z., Li, J., Chen, H., Wu, Y., Fu, T., Zhu, H. and Saand, M.A. 2021. Chromosome‐scale genome assembly of areca palm (Areca catechu). Molecular ecology resources, 21(7), pp.2504-2519. | <https://onlinelibrary.wiley.com/doi/10.1111/1755-0998.13446> |
| Arecaceae | *Areca catechu* | 69.19 | 61.68 | NA | NA | 1.05 | NA | Zhou, G., Yin, H., Chen, F., Wang, Y., Gao, Q., Yang, F., He, C., Zhang, L. and Wan, Y. 2022. The genome of Areca catechu provides insights into sex determination of monoecious plants. New Phytologist, 236(6), pp.2327-2343. | <https://nph.onlinelibrary.wiley.com/doi/10.1111/nph.18471> |
| Arecaceae | *Cocos nucifera* | 74.48 | 67.10 | NA | NA | 0.87 | 2.64 | Xiao, Y., Xu, P., Fan, H., Baudouin, L., Xia, W., Bocs, S., Xu, J., Li, Q., Guo, A., Zhou, L. and Li, J. 2017. The genome draft of coconut (Cocos nucifera). Gigascience, 6(11), p.gix095. | <https://academic.oup.com/gigascience/article/6/11/gix095/4345653> |
| Arecaceae | *Cocos nucifera* | 78.33 | 60.26 | 27.27 | 20.77 | 0.34 | 7.67 | Lantican, D.V., Strickler, S.R., Canama, A.O., Gardoce, R.R., Mueller, L.A. and Galvez, H.F. 2019. De novo genome sequence assembly of dwarf coconut (Cocos nucifera L.‘Catigan Green Dwarf’) provides insights into genomic variation between coconut types and related palm species. G3: Genes, Genomes, Genetics, 9(8), pp.2377-2393. | <https://academic.oup.com/g3journal/article/9/8/2377/6026782> |
| Arecaceae | *Cocos nucifera* | 77.29 | 58.85 | 36.80 | 21.44 | NA | NA | Muliyar, R.K., Chowdappa, P., Behera, S.K., Kasaragod, S., Gangaraj, K.P., Kotimoole, C.N., Nekrakalaya, B., Mohanty, V., Sampgod, R.B., Banerjee, G. and Das, A.J. 2020. Assembly and annotation of the nuclear and organellar genomes of a dwarf coconut (Chowghat Green Dwarf) possessing enhanced disease resistance. OMICS: A Journal of Integrative Biology, 24(12), pp.726-742. | <https://www.liebertpub.com/doi/10.1089/omi.2020.0147> |
| Arecaceae | *Elaeis guineensis* | NA | 54.00 | 33.00 | 8.00 | NA | 2.00 | Singh, R., Ong-Abdullah, M., Low, E.T.L., Manaf, M.A.A., Rosli, R., Nookiah, R., Ooi, L.C.L., Ooi, S.E., Chan, K.L., Halim, M.A. and Azizi, N. 2013. Oil palm genome sequence reveals divergence of interfertile species in Old and New worlds. Nature, 500(7462), pp.335-339. | <https://www.nature.com/articles/nature12309> |
| Arecaceae | *Elaeis guineensis* | 73.70 | 55.79 | 39.46 | 17.19 | NA | NA | Wang, L., Lee, M., Wan, Z.Y., Bai, B., Ye, B., Alfiko, Y., Rahmadsyah, R., Purwantomo, S., Song, Z., Suwanto, A. and Yue, G.H. 2022. Chromosome-level reference genome provides insights into divergence and stress adaptation of the African oil palm. Genomics, Proteomics & Bioinformatics. | <https://www.sciencedirect.com/science/article/pii/S1672022922001437?via%3Dihub> |
| Arecaceae | *Phoenix dactylifera* | 38.41 | 21.99 | 14.03 | 4.17 | 0.46 | 0.32 | Al-Mssallem, I.S., Hu, S., Zhang, X., Lin, Q., Liu, W., Tan, J., Yu, X., Liu, J., Pan, L., Zhang, T. and Yin, Y. 2013. Genome sequence of the date palm Phoenix dactylifera L. Nature communications, 4(1), p.2274. | <https://www.nature.com/articles/ncomms3274> |
| Arecaceae | *Phoenix dactylifera* | 52.25 | NA | 8.78 | 6.35 | 2.58 | 1.93 | Hazzouri, K.M., Gros-Balthazard, M., Flowers, J.M., Copetti, D., Lemansour, A., Lebrun, M., Masmoudi, K., Ferrand, S., Dhar, M.I., Fresquez, Z.A. and Rosas, U. 2019. Genome-wide association mapping of date palm fruit traits. Nature Communications, 10(1), p.4680. | <https://www.nature.com/articles/s41467-019-12604-9> |
| Arecaceae | *Calamus simplicifolius* | 54.15 | 47.85 | NA | NA | 3.77 | 4.23 | Zhao, H., Wang, S., Wang, J., Chen, C., Hao, S., Chen, L., Fei, B., Han, K., Li, R., Shi, C. and Sun, H. 2018. The chromosome-level genome assemblies of two rattans (Calamus simplicifolius and Daemonorops jenkinsiana). Gigascience, 7(9), p.giy097. | <https://academic.oup.com/gigascience/article/7/9/giy097/5067873> |
| Arecaceae | *Daemonorops jenkinsiana* | 70.00 | 61.09 | NA | NA | 4.62 | 5.82 | Zhao, H., Wang, S., Wang, J., Chen, C., Hao, S., Chen, L., Fei, B., Han, K., Li, R., Shi, C. and Sun, H. 2018. The chromosome-level genome assemblies of two rattans (Calamus simplicifolius and Daemonorops jenkinsiana). Gigascience, 7(9), p.giy097. | <https://academic.oup.com/gigascience/article/7/9/giy097/5067873> |
| Arecaceae | *Korthalsia laciniosa* | 10.49 | 5.54 | NA | NA | 1.40 | 0.65 | Dasgupta, M.G., Dev, S.A., Parveen, A.B.M., Sarath, P. and Sreekumar, V.B. 2021. Draft genome of Korthalsia laciniosa (Griff.) Mart., a climbing rattan elucidates its phylogenetic position. Genomics, 113(4), pp.2010-2022. | <https://www.sciencedirect.com/science/article/pii/S0888754321001506?via%3Dihub> |
| Arecaceae | *Nypa fruticans* | 38.05 | 23.59 | NA | NA | 0.91 | 2.89 | He, Z., Feng, X., Chen, Q., Li, L., Li, S., Han, K., Guo, Z., Wang, J., Liu, M., Shi, C. and Xu, S. 2022. Evolution of coastal forests based on a full set of mangrove genomes. Nature ecology & evolution, 6(6), pp.738-749. | <https://www.nature.com/articles/s41559-022-01744-9> |
| Asparagaceae | *Asparagus setaceus* | 65.59 | 42.51 | 8.08 | 19.50 | 2.90 | 4.12 | Li, S.F., Wang, J., Dong, R., Zhu, H.W., Lan, L.N., Zhang, Y.L., Li, N., Deng, C.L. and Gao, W.J. 2020. Chromosome-level genome assembly, annotation and evolutionary analysis of the ornamental plant Asparagus setaceus. Horticulture Research, 7. | http://dx.doi.org/10.1038/s41438-020-0271-y |
| Asparagaceae | *Dracaena cambodiana* | 53.45 | 26.13 | 2.97 | 22.31 | 2.56 | 2.85 | Ding, X., Mei, W., Huang, S., Wang, H., Zhu, J., Hu, W., Ding, Z., Tie, W., Peng, S. and Dai, H. 2018. Genome survey sequencing for the characterization of genetic background of Dracaena cambodiana and its defense response during dragon’s blood formation. PLoS One, 13(12), p.e0209258. | http://dx.doi.org/10.1371/journal.pone.0209258 |
| Asparagaceae | *Dracaena cochinchinensis* | 60.15 | 51.06 | 5.90 | 41.00 | 3.43 | 4.05 | Xu, Y., Zhang, K., Zhang, Z., Liu, Y., Lv, F., Sun, P., Gao, S., Wang, Q., Yu, C., Jiang, J. and Li, C. 2022. A chromosome-level genome assembly for Dracaena cochinchinensis reveals the molecular basis of its longevity and formation of dragon’s blood. Plant Communications, 3(6). | http://dx.doi.org/10.1016/j.xplc.2022.100456 |
| Asphodelaceae | *Aloe vera* | 82.26 | 26.71 | 7.34 | 19.37 | NA | 0.13 | Jaiswal, S.K., Mahajan, S., Chakraborty, A., Kumar, S. and Sharma, V.K. 2021. The genome sequence of Aloe vera reveals adaptive evolution of drought tolerance mechanisms. Iscience, 24(2). | http://dx.doi.org/10.1016/j.isci.2021.102079 |
| Asphodelaceae | *Hemerocallis citrina* | 86.20 | 62.40 | NA | NA | 6.63 | 12.27 | Qing, Z., Liu, J., Yi, X., Liu, X., Hu, G., Lao, J., He, W., Yang, Z., Zou, X., Sun, M. and Huang, P. 2021. The chromosome-level Hemerocallis citrina Borani genome provides new insights into the rutin biosynthesis and the lack of colchicine. Horticulture Research, 8, p.89. | http://dx.doi.org/10.1038/s41438-021-00539-6 |
| Bromeliaceae | *Aechmea fasciata* | 61.72 | 33.00 | 3.97 | 23.92 | 0.96 | 0.70 | Li, Z., Wang, J., Zhang, X., Zhu, G., Fu, Y., Jing, Y., Huang, B., Wang, X., Meng, C., Yang, Q. and Xu, L. 2022. The genome of Aechmea fasciata provides insights into the evolution of tank epiphytic habits and ethylene-induced flowering. Communications Biology, 5(1), p.920. | <https://www.nature.com/articles/s42003-022-03918-4> |
| Bromeliaceae | *Ananas bracteatus* | 74.66 | 35.98 | 4.46 | 17.05 | 7.23 | 22.12 | Chen, L.Y., VanBuren, R., Paris, M., Zhou, H., Zhang, X., Wai, C.M., Yan, H., Chen, S., Alonge, M., Ramakrishnan, S. and Liao, Z. 2019. The bracteatus pineapple genome and domestication of clonally propagated crops. Nature Genetics, 51(10), pp.1549-1558. | <https://www.nature.com/articles/s41588-019-0506-8> |
| Bromeliaceae | *Ananas comosus* | 68.20 | 44.80 | 8.23 | 21.29 | 2.57 | 13.18 | Feng, L., Wang, J., Mao, M., Yang, W., Adje, M.O., Xue, Y., Zhou, X., Zhang, H., Luo, J., Tang, R. and Tan, L. 2022. The highly continuous reference genome of a leaf-chimeric red pineapple (Ananas comosus var. bracteatus f. tricolor) provides insights into elaboration of leaf color. G3, 12(2), p.jkab452. | <https://academic.oup.com/g3journal/article/12/2/jkab452/6501447> |
| Bromeliaceae | *Puya raimondii* | 67.02 | 47.30 | 12.32 | 18.73 | NA | 0.07 | Liu, L., Tumi, L., Suni, M.L., Arakaki, M., Wang, Z.F. and Ge, X.J. 2021. Draft genome of Puya raimondii (Bromeliaceae), the Queen of the Andes. Genomics, 113(4), pp.2537-2546. | <https://www.sciencedirect.com/science/article/pii/S0888754321002160?via%3Dihub> |
| Cannaceae | *Canna edulis* | 62.62 | 22.80 | 8.20 | 7.65 | 3.46 | 9.63 | Fu, Y., Jiang, S., Zou, M., Xiao, J., Yang, L., Luo, C., Rao, P., Wang, W., Ou, Z., Liu, F. and Xia, Z. 2022. High-quality reference genome sequences of two Cannaceae species provide insights into the evolution of Cannaceae. Frontiers in Plant Science, 13. | <https://www.frontiersin.org/articles/10.3389/fpls.2022.955904/full> |
| Cannaceae | *Canna indica* | 63.60 | 24.15 | 9.25 | 6.05 | 3.26 | 10.59 | Fu, Y., Jiang, S., Zou, M., Xiao, J., Yang, L., Luo, C., Rao, P., Wang, W., Ou, Z., Liu, F. and Xia, Z. 2022. High-quality reference genome sequences of two Cannaceae species provide insights into the evolution of Cannaceae. Frontiers in Plant Science, 13. | <https://www.frontiersin.org/articles/10.3389/fpls.2022.955904/full> |
| Cannaceae | *Canna indica* | 58.72 | 33.83 | 15.84 | 17.46 | NA | 18.87 | Yang, P., Ling, X.Y., Zhou, X.F., Chen, Y.X., Wang, T.T., Lin, X.J., Zhao, Y.Y., Ye, Y.S., Huang, L.X., Sun, Y.W. and Qi, Y.X. 2023. Comparing genomes of Fructus Amomi-producing species reveals genetic basis of volatile terpenoid divergence. Plant Physiology, p.kiad400. | <https://academic.oup.com/plphys/advance-article/doi/10.1093/plphys/kiad400/7222149> |
| Cyperaceae | *Carex cristatella* | 40.04 | 6.86 | 4.27 | 2.43 | 1.37 | 9.97 | Planta, J., Liang, Y.Y., Xin, H., Chansler, M.T., Prather, L.A., Jiang, N., Jiang, J. and Childs, K.L. 2022. Chromosome-scale genome assemblies and annotations for Poales species Carex cristatella, Carex scoparia, Juncus effusus, and Juncus inflexus. G3, 12(10), p.jkac211. | <https://academic.oup.com/g3journal/article/12/10/jkac211/6670624> |
| Cyperaceae | *Carex kokanica* | 52.47 | 23.84 | NA | NA | 6.77 | 26.10 | Qu, G., Bao, Y., Liao, Y., Liu, C., Zi, H., Bai, M., Liu, Y., Tu, D., Wang, L., Chen, S. and Zhou, G. 2022. Draft genomes assembly and annotation of Carex parvula and Carex kokanica reveals stress-specific genes. Scientific reports, 12(1), p.4970. | <https://www.nature.com/articles/s41598-022-08783-z> |
| Cyperaceae | *Carex littledalei* | 53.93 | 27.87 | NA | NA | 5.42 | 18.53 | Can, M., Wei, W., Zi, H., Bai, M., Liu, Y., Gao, D., Tu, D., Bao, Y., Wang, L., Chen, S. and Zhao, X. 2020. Genome sequence of Kobresia littledalei, the first chromosome-level genome in the family Cyperaceae. Sci Data 7: 175. | <https://www.nature.com/articles/s41597-020-0518-3> |
| Cyperaceae | *Carex myosuroides* | 51.89 | 21.68 | 7.55 | 8.66 | 2.27 | 20.74 | Ning, Y., Li, Y., Dong, S.B., Yang, H.G., Li, C.Y., Xiong, B., Yang, J., Hu, Y.K., Mu, X.Y. and Xia, X.F. 2023. The chromosome-scale genome of Kobresia myosuroides sheds light on karyotype evolution and recent diversification of a dominant herb group on the Qinghai-Tibet Plateau. DNA Research, 30(1), p.dsac049. | <https://academic.oup.com/dnaresearch/article/30/1/dsac049/6887608> |
| Cyperaceae | *Carex parvula* | 47.97 | 25.47 | NA | NA | 5.34 | 18.60 | Qu, G., Bao, Y., Liao, Y., Liu, C., Zi, H., Bai, M., Liu, Y., Tu, D., Wang, L., Chen, S. and Zhou, G. 2022. Draft genomes assembly and annotation of Carex parvula and Carex kokanica reveals stress-specific genes. Scientific reports, 12(1), p.4970. | <https://www.nature.com/articles/s41598-022-08783-z> |
| Cyperaceae | *Carex scoparia* | 39.93 | 6.32 | 3.68 | 2.28 | 1.24 | 11.68 | Planta, J., Liang, Y.Y., Xin, H., Chansler, M.T., Prather, L.A., Jiang, N., Jiang, J. and Childs, K.L. 2022. Chromosome-scale genome assemblies and annotations for Poales species Carex cristatella, Carex scoparia, Juncus effusus, and Juncus inflexus. G3, 12(10), p.jkac211. | <https://academic.oup.com/g3journal/article/12/10/jkac211/6670624> |
| Cyperaceae | *Cyperus esculentus* | 33.90 | 8.67 | NA | NA | 1.79 | 5.02 | Zhao, X., Yi, L., Ren, Y., Li, J., Ren, W., Hou, Z., Su, S., Wang, J., Zhang, Y., Dong, Q. and Yang, X. 2023. Chromosome-scale genome assembly of the yellow nutsedge (Cyperus esculentus). Genome Biology and Evolution, 15(3), p.evad027. | <https://academic.oup.com/gbe/article/15/3/evad027/7049323> |
| Dioscoreaceae | *Dioscorea alata* | 66.82 | 32.00 | 9.35 | 15.47 | 2.31 | 1.50 | Bredeson, J.V., Lyons, J.B., Oniyinde, I.O., Okereke, N.R., Kolade, O., Nnabue, I., Nwadili, C.O., Hřibová, E., Parker, M., Nwogha, J. and Shu, S. 2022. Chromosome evolution and the genetic basis of agronomically important traits in greater yam. Nature communications, 13(1), p.2001. | http://dx.doi.org/10.1038/s41467-022-29114-w |
| Dioscoreaceae | *Dioscorea zingiberensis* | 60.17 | 47.87 | 4.56 | 20.46 | 6.93 | 8.62 | Li, Y., Tan, C., Li, Z., Guo, J., Li, S., Chen, X., Wang, C., Dai, X., Yang, H., Song, W. and Hou, L. 2022. The genome of Dioscorea zingiberensis sheds light on the biosynthesis, origin and evolution of the medicinally important diosgenin saponins. Horticulture Research, 9, p.uhac165. | http://dx.doi.org/10.1093/hr/uhac165 |
| Juncaceae | *Juncus effusus* | 48.64 | 14.95 | 10.39 | 4.39 | 1.98 | 3.82 | Planta, J., Liang, Y.Y., Xin, H., Chansler, M.T., Prather, L.A., Jiang, N., Jiang, J. and Childs, K.L. 2022. Chromosome-scale genome assemblies and annotations for Poales species Carex cristatella, Carex scoparia, Juncus effusus, and Juncus inflexus. G3, 12(10), p.jkac211. | <https://academic.oup.com/g3journal/article/12/10/jkac211/6670624> |
| Juncaceae | *Juncus inflexus* | 42.73 | 15.62 | 10.39 | 5.04 | 1.94 | 5.41 | Planta, J., Liang, Y.Y., Xin, H., Chansler, M.T., Prather, L.A., Jiang, N., Jiang, J. and Childs, K.L. 2022. Chromosome-scale genome assemblies and annotations for Poales species Carex cristatella, Carex scoparia, Juncus effusus, and Juncus inflexus. G3, 12(10), p.jkac211. | <https://academic.oup.com/g3journal/article/12/10/jkac211/6670624> |
| Marantaceae | *Thalia dealbata* | 39.34 | 12.36 | NA | NA | 1.17 | 2.09 | Tang, M., Huang, J., Ma, X., Du, J., Bi, Y., Guo, P., Lu, H. and Wang, L. 2023. A near-complete genome assembly of Thalia dealbata Fraser (Marantaceae). Frontiers in Plant Science, 14, p.1183361. | <https://www.frontiersin.org/articles/10.3389/fpls.2023.1183361/full> |
| Musaceae | *Ensete glaucum* | 55.02 | 37.20 | 17.64 | 19.25 | 0.70 | 7.18 | Wang, Z., Rouard, M., Biswas, M.K., Droc, G., Cui, D., Roux, N., Baurens, F.C., Ge, X.J., Schwarzacher, T., Heslop-Harrison, P.J. and Liu, Q. 2022. A chromosome-level reference genome of Ensete glaucum gives insight into diversity and chromosomal and repetitive sequence evolution in the Musaceae. GigaScience, 11. | <https://academic.oup.com/gigascience/article/doi/10.1093/gigascience/giac027/6576245> |
| Musaceae | *Ensete glaucum* | 45.17 | 33.01 | 13.92 | 11.79 | NA | 1.31 | Wang, Z.F., Rouard, M., Droc, G., Heslop-Harrison, P. and Ge, X.J. 2023. Genome assembly of Musa beccarii shows extensive chromosomal rearrangements and genome expansion during evolution of Musaceae genomes. GigaScience, 12, p.giad005. | <https://academic.oup.com/gigascience/article/doi/10.1093/gigascience/giad005/7049365> |
| Musaceae | *Ensete ventricosum* | 32.65 | NA | NA | NA | NA | NA | Harrison, J., Moore, K.A., Paszkiewicz, K., Jones, T., Grant, M.R., Ambacheew, D., Muzemil, S. and Studholme, D.J. 2014. A draft genome sequence for Ensete ventricosum, the drought-tolerant “tree against hunger”. Agronomy, 4(1), pp.13-33. | <https://www.mdpi.com/2073-4395/4/1/13> |
| Musaceae | *Musa acuminata* | 53.55 | 40.03 | 28.86 | 10.98 | 2.03 | 1.93 | Wang, Z., Rouard, M., Biswas, M.K., Droc, G., Cui, D., Roux, N., Baurens, F.C., Ge, X.J., Schwarzacher, T., Heslop-Harrison, P.J. and Liu, Q. 2022. A chromosome-level reference genome of Ensete glaucum gives insight into diversity and chromosomal and repetitive sequence evolution in the Musaceae. GigaScience, 11. | <https://academic.oup.com/gigascience/article/doi/10.1093/gigascience/giac027/6576245> |
| Musaceae | *Musa acuminata* | 41.85 | NA | 20.36 | 14.92 | 0.81 | 2.03 | Wang, Z., Miao, H., Liu, J., Xu, B., Yao, X., Xu, C., Zhao, S., Fang, X., Jia, C., Wang, J. and Zhang, J. 2019. Musa balbisiana genome reveals subgenome evolution and functional divergence. Nature plants, 5(8), pp.810-821. | <https://www.nature.com/articles/s41477-019-0452-6> |
| Musaceae | *Musa acuminata* | 46.70 | 39.45 | 29.48 | 6.11 | NA | 1.06 | Wang, Z.F., Rouard, M., Droc, G., Heslop-Harrison, P. and Ge, X.J. 2023. Genome assembly of Musa beccarii shows extensive chromosomal rearrangements and genome expansion during evolution of Musaceae genomes. GigaScience, 12, p.giad005. | <https://academic.oup.com/gigascience/article/doi/10.1093/gigascience/giad005/7049367> |
| Musaceae | *Musa acuminata* | 35.43 | 32.72 | 15.62 | 18.34 | 0.95 | 2.13 | Wu, W., Yang, Y.L., He, W.M., Rouard, M., Li, W.M., Xu, M., Roux, N. and Ge, X.J. 2016. Whole genome sequencing of a banana wild relative Musa itinerans provides insights into lineage-specific diversification of the Musa genus. Scientific reports, 6(1), pp.1-11. | <https://www.nature.com/articles/srep31586> |
| Musaceae | Musa acuminata V2 | 38.38 | 26.38 | 8.96 | 4.67 | 2.74 | 2.18 | Belser, C., Baurens, F.C., Noel, B., Martin, G., Cruaud, C., Istace, B., Yahiaoui, N., Labadie, K., Hřibová, E., Doležel, J. and Lemainque, A. 2021. Telomere-to-telomere gapless chromosomes of banana using nanopore sequencing. Communications biology, 4(1), p.1047. | <https://www.nature.com/articles/s42003-021-02559-3> |
| Musaceae | *Musa balbisiana* | 49.35 | 34.12 | 12.43 | 5.14 | 2.94 | 2.15 | Belser, C., Baurens, F.C., Noel, B., Martin, G., Cruaud, C., Istace, B., Yahiaoui, N., Labadie, K., Hřibová, E., Doležel, J. and Lemainque, A. 2021. Telomere-to-telomere gapless chromosomes of banana using nanopore sequencing. Communications biology, 4(1), p.1047. | <https://www.nature.com/articles/s42003-021-02559-3> |
| Musaceae | *Musa balbisiana* | 55.75 | NA | 28.04 | 12.88 | 1.30 | 2.12 | Wang, Z., Miao, H., Liu, J., Xu, B., Yao, X., Xu, C., Zhao, S., Fang, X., Jia, C., Wang, J. and Zhang, J. 2019. Musa balbisiana genome reveals subgenome evolution and functional divergence. Nature plants, 5(8), pp.810-821. | <https://www.nature.com/articles/s41477-019-0452-6> |
| Musaceae | *Musa balbisiana* | 41.53 | 35.59 | 25.90 | 6.32 | NA | 1.35 | Wang, Z.F., Rouard, M., Droc, G., Heslop-Harrison, P. and Ge, X.J. 2023. Genome assembly of Musa beccarii shows extensive chromosomal rearrangements and genome expansion during evolution of Musaceae genomes. GigaScience, 12, p.giad005. | <https://academic.oup.com/gigascience/article/doi/10.1093/gigascience/giad005/7049364> |
| Musaceae | *Musa beccarii* | 51.79 | 43.47 | 25.35 | 9.07 | NA | 2.79 | Wang, Z.F., Rouard, M., Droc, G., Heslop-Harrison, P. and Ge, X.J. 2023. Genome assembly of Musa beccarii shows extensive chromosomal rearrangements and genome expansion during evolution of Musaceae genomes. GigaScience, 12, p.giad005. | <https://academic.oup.com/gigascience/article/doi/10.1093/gigascience/giad005/7049363> |
| Musaceae | *Musa itinerans* | 32.12 | 15.89 | 7.30 | 4.37 | NA | 2.85 | Wang, Z.F., Rouard, M., Droc, G., Heslop-Harrison, P. and Ge, X.J. 2023. Genome assembly of Musa beccarii shows extensive chromosomal rearrangements and genome expansion during evolution of Musaceae genomes. GigaScience, 12, p.giad005. | <https://academic.oup.com/gigascience/article/doi/10.1093/gigascience/giad005/7049365> |
| Musaceae | *Musa itinerans* | 38.95 | 34.07 | 16.70 | 16.25 | 1.45 | 3.15 | Wu, W., Yang, Y.L., He, W.M., Rouard, M., Li, W.M., Xu, M., Roux, N. and Ge, X.J. 2016. Whole genome sequencing of a banana wild relative Musa itinerans provides insights into lineage-specific diversification of the Musa genus. Scientific reports, 6(1), pp.1-11. | <https://www.nature.com/articles/srep31586> |
| Musaceae | *Musa schizocarpa* | 56.34 | 39.38 | 13.77 | 5.85 | 3.48 | 1.74 | Belser, C., Baurens, F.C., Noel, B., Martin, G., Cruaud, C., Istace, B., Yahiaoui, N., Labadie, K., Hřibová, E., Doležel, J. and Lemainque, A. 2021. Telomere-to-telomere gapless chromosomes of banana using nanopore sequencing. Communications biology, 4(1), p.1047. | <https://www.nature.com/articles/s42003-021-02559-3> |
| Musaceae | *Musa schizocarpa* | 52.26 | 43.00 | 30.06 | 7.23 | NA | 0.83 | Wang, Z.F., Rouard, M., Droc, G., Heslop-Harrison, P. and Ge, X.J. 2023. Genome assembly of Musa beccarii shows extensive chromosomal rearrangements and genome expansion during evolution of Musaceae genomes. GigaScience, 12, p.giad005. | <https://academic.oup.com/gigascience/article/doi/10.1093/gigascience/giad005/7049366> |
| Musaceae | *Musa textilis* Née var. Abuab | 53.60 | NA | 34.46 | 10.03 | 0.92 | 2.13 | Galvez, L.C., Koh, R.B.L., Barbosa, C.F.C., Asunto, J.C., Catalla, J.L., Atienza, R.G., Costales, K.T., Aquino, V.M. and Zhang, D. 2021. Sequencing and de novo assembly of abaca (Musa textilis Née) var. Abuab genome. Genes, 12(8), p.1202. | <https://www.mdpi.com/2073-4425/12/8/1202> |
| Musaceae | *Musa troglodytarum* | 60.83 | 51.48 | 36.40 | 15.10 | 0.55 | 0.41 | Li, Z., Wang, J., Fu, Y., Jing, Y., Huang, B., Chen, Y., Wang, Q., Wang, X.B., Meng, C., Yang, Q. and Xu, L. 2022. The Musa troglodytarum L. genome provides insights into the mechanism of non-climacteric behaviour and enrichment of carotenoids. BMC biology, 20(1), p.186. | <https://bmcbiol.biomedcentral.com/articles/10.1186/s12915-022-01391-3> |
| Musaceae | *Musa acuminata* | 46.41 | 39.97 | 30.09 | 9.28 | NA | 5.48 | Yang, P., Ling, X.Y., Zhou, X.F., Chen, Y.X., Wang, T.T., Lin, X.J., Zhao, Y.Y., Ye, Y.S., Huang, L.X., Sun, Y.W. and Qi, Y.X. 2023. Comparing genomes of Fructus Amomi-producing species reveals genetic basis of volatile terpenoid divergence. Plant Physiology, p.kiad400. | <https://academic.oup.com/plphys/advance-article/doi/10.1093/plphys/kiad400/7222150> |
| Orchidaceae | *Apostasia shenzhenica* | 42.05 | 22.06 | 4.97 | 11.84 | 12.68 | 6.46 | Zhang, G.Q., Liu, K.W., Li, Z., Lohaus, R., Hsiao, Y.Y., Niu, S.C., Wang, J.Y., Lin, Y.C., Xu, Q., Chen, L.J. and Yoshida, K. 2017. The Apostasia genome and the evolution of orchids. Nature, 549(7672), pp.379-383. | http://dx.doi.org/10.1038/nature23897 |
| Orchidaceae | *Cremastra appendiculata* | 59.15 | 56.25 | NA | NA | 1.38 | 2.70 | Wang, J., Xie, J., Chen, H., Qiu, X., Cui, H., Liu, Y., Sahu, S.K., Fang, D., Li, T., Wang, M. and Chen, Y. 2022. A draft genome of the medicinal plant Cremastra appendiculata (D. Don) provides insights into the colchicine biosynthetic pathway. Communications Biology, 5(1), p.1294. | http://dx.doi.org/10.1038/s42003-022-04229-4 |
| Orchidaceae | *Cymbidium ensifolium* | 80.58 | 48.98 | NA | NA | 16.46 | 15.08 | Ai, Y., Li, Z., Sun, W.H., Chen, J., Zhang, D., Ma, L., Zhang, Q.H., Chen, M.K., Zheng, Q.D., Liu, J.F. and Jiang, Y.T. 2021. The Cymbidium genome reveals the evolution of unique morphological traits. Horticulture research, 8. | http://dx.doi.org/10.1038/s41438-021-00683-z |
| Orchidaceae | *Dendrobium officinale* | 63.33 | 22.23 | NA | NA | 8.17 | 4.59 | Yan, L., Wang, X., Liu, H., Tian, Y., Lian, J., Yang, R., Hao, S., Wang, X., Yang, S., Li, Q. and Qi, S. 2015. The genome of Dendrobium officinale illuminates the biology of the important traditional Chinese orchid herb. Molecular plant, 8(6), pp.922-934. | http://dx.doi.org/10.1016/j.molp.2014.12.011 |
| Orchidaceae | *Phalaenopsis equestris* | 61.53 | 46.47 | 6.95 | 39.66 | 7.70 | 4.63 | Cai, J., Liu, X., Vanneste, K., Proost, S., Tsai, W.C., Liu, K.W., Chen, L.J., He, Y., Xu, Q., Bian, C. and Zheng, Z. 2015. The genome sequence of the orchid Phalaenopsis equestris. Nature genetics, 47(1), pp.65-72. | http://dx.doi.org/10.1038/ng.3149 |
| Orchidaceae | *Vanilla planifolia* | 44.30 | 10.00 | NA | NA | 10.80 | 17.00 | Hasing, T., Tang, H., Brym, M., Khazi, F., Huang, T. and Chambers, A.H. 2020. A phased Vanilla planifolia genome enables genetic improvement of flavour and production. Nature Food, 1(12), pp.811-819. | http://dx.doi.org/10.1038/s43016-020-00197-2 |
| Poaceae | *Streptochaeta angustifolia* | 66.82 | 42.90 | 8.90 | 28.16 | NA | 23.39 | Seetharam, A.S., Yu, Y., Bélanger, S., Clark, L.G., Meyers, B.C., Kellogg, E.A. and Hufford, M.B. 2021. The Streptochaeta genome and the evolution of the grasses. Frontiers in Plant Science, 12, p.710383. | http://dx.doi.org/10.3389/fpls.2021.710383 |
| Poaceae | *Phragmites australis* | 56.19 | 36.42 | NA | NA | 1.74 | 11.43 | Oh, D.H., Kowalski, K.P., Quach, Q.N., Wijesinghege, C., Tanford, P., Dassanayake, M. and Clay, K. 2022. Novel genome characteristics contribute to the invasiveness of Phragmites australis (common reed). Molecular Ecology, 31(4), pp.1142-1159. | http://dx.doi.org/10.1111/mec.16293 |
| Poaceae | *Raddia guianensis* | 54.15 | 29.86 | 9.01 | 20.85 | 2.37 | 6.01 | Guo, Z.H., Ma, P.F., Yang, G.Q., Hu, J.Y., Liu, Y.L., Xia, E.H., Zhong, M.C., Zhao, L., Sun, G.L., Xu, Y.X. and Zhao, Y.J. 2019. Genome sequences provide insights into the reticulate origin and unique traits of woody bamboos. Molecular plant, 12(10), pp.1353-1365. | http://dx.doi.org/10.1016/j.molp.2019.05.009 |
| Poaceae | *Cynodon dactylon* | 37.91 | 22.79 | 6.38 | 9.44 | 4.95 | 4.28 | Zhang, B., Chen, S., Liu, J., Yan, Y.B., Chen, J., Li, D. and Liu, J.Y. 2022. A high-quality haplotype-resolved genome of common bermudagrass (Cynodon dactylon L.) provides insights into polyploid genome stability and prostrate growth. Frontiers in Plant Science, 13, p.890980. | http://dx.doi.org/10.3389/fpls2022.890980 |
| Poaceae | *Eleusine indica* | 21.90 | 13.80 | NA | NA | 1.40 | 2.90 | Zhang, H., Hall, N., Goertzen, L.R., Bi, B., Chen, C.Y., Peatman, E., Lowe, E.K., Patel, J. and McElroy, J.S. 2019. Development of a goosegrass (Eleusine indica) draft genome and application to weed science research. Pest management science, 75(10), pp.2776-2784. | http://dx.doi.org/10.1002/ps.5389 |
| Poaceae | *Eragrostis curvula* | 28.70 | 16.97 | 3.14 | 13.62 | NA | 4.00 | Carballo, J., Santos, B.A.C.M., Zappacosta, D., Garbus, I., Selva, J.P., Gallo, C.A., Díaz, A., Albertini, E., Caccamo, M. and Echenique, V. 2019. A high-quality genome of Eragrostis curvula grass provides insights into Poaceae evolution and supports new strategies to enhance forage quality. Scientific Reports, 9(1), p.10250. | http://dx.doi.org/10.1038/s41598-019-46610-0 |
| Poaceae | *Leersia perrieri* | 26.83 | NA | 3.91 | 8.32 | 2.04 | 10.07 | Stein, J.C., Yu, Y., Copetti, D., Zwickl, D.J., Zhang, L., Zhang, C., Chougule, K., Gao, D., Iwata, A., Goicoechea, J.L. and Wei, S. 2018. Genomes of 13 domesticated and wild rice relatives highlight genetic conservation, turnover and innovation across the genus Oryza. Nature genetics, 50(2), pp.285-296. | <https://www.nature.com/articles/s41588-018-0040-0#Tab1> |
| Poaceae | *Oryza sativa* | 30.78 | 19.85 | 3.08 | 16.39 | 1.11 | 5.82 | Jia, J., Zhao, S., Kong, X., Li, Y., Zhao, G., He, W., Appels, R., Pfeifer, M., Tao, Y., Zhang, X. and Jing, R. 2013. Aegilops tauschii draft genome sequence reveals a gene repertoire for wheat adaptation. Nature, 496(7443), pp.91-95. | http://dx.doi.org/10.1038/nature12028 |
| Poaceae | *Oryza sativa* | 48.70 | 21.60 | 3.30 | 17.70 | 1.00 | 13.70 | Tanaka, H., Hirakawa, H., Kosugi, S., Nakayama, S., Ono, A., Watanabe, A., Hashiguchi, M., Gondo, T., Ishigaki, G., Muguerza, M. and Shimizu, K. 2016. Sequencing and comparative analyses of the genomes of zoysiagrasses. DNA Research, 23(2), pp.171-180. | http://dx.doi.org/10.1093/dnares/dsw006 |
| Poaceae | *Oryza sativa* | 32.02 | 23.49 | 3.45 | 18.55 | 1.05 | 8.53 | Jain, R., Jenkins, J., Shu, S., Chern, M., Martin, J.A., Copetti, D., Duong, P.Q., Pham, N.T., Kudrna, D.A., Talag, J. and Schackwitz, W.S. 2019. Genome sequence of the model rice variety KitaakeX. BMC genomics, 20, pp.1-9. | <https://bmcgenomics.biomedcentral.com/articles/10.1186/s12864-019-6262-4> |
| Poaceae | *Oryza sativa* | 49.04 | NA | 4.08 | 21.89 | 1.51 | 20.80 | Stein, J.C., Yu, Y., Copetti, D., Zwickl, D.J., Zhang, L., Zhang, C., Chougule, K., Gao, D., Iwata, A., Goicoechea, J.L. and Wei, S. 2018. Genomes of 13 domesticated and wild rice relatives highlight genetic conservation, turnover and innovation across the genus Oryza. Nature genetics, 50(2), pp.285-296. | <https://www.nature.com/articles/s41588-018-0040-0#Tab1> |
| Poaceae | *Alloteropsis semialata* | 51.00 | NA | 4.63 | 31.05 | NA | 3.44 | Dunning, L.T., Olofsson, J.K., Parisod, C., Choudhury, R.R., Moreno-Villena, J.J., Yang, Y., Dionora, J., Quick, W.P., Park, M., Bennetzen, J.L. and Besnard, G. 2019. Lateral transfers of large DNA fragments spread functional genes among grasses. Proceedings of the National Academy of Sciences, 116(10), pp.4416-4425. | http://dx.doi.org/10.1073/pnas.1810031116 |
| Poaceae | *Saccharum spontaneum* | 57.52 | 40.64 | 11.47 | 28.87 | 1.55 | 8.45 | Zhang, J., Zhang, X., Tang, H., Zhang, Q., Hua, X., Ma, X., Zhu, F., Jones, T., Zhu, X., Bowers, J. and Wai, C.M. 2018. Allele-defined genome of the autopolyploid sugarcane Saccharum spontaneum L. Nature genetics, 50(11), pp.1565-1573. | http://dx.doi.org/10.1038/s41588-018-0237-2 |
| Poaceae | *Sorghum bicolor* | 65.83 | 49.70 | 6.81 | 42.85 | 0.98 | 7.17 | Jia, J., Zhao, S., Kong, X., Li, Y., Zhao, G., He, W., Appels, R., Pfeifer, M., Tao, Y., Zhang, X. and Jing, R. 2013. Aegilops tauschii draft genome sequence reveals a gene repertoire for wheat adaptation. Nature, 496(7443), pp.91-95. | http://dx.doi.org/10.1038/nature12028 |
| Poaceae | *Sorghum bicolor* | 64.80 | 47.90 | 6.50 | 41.40 | 1.40 | 9.10 | Tanaka, H., Hirakawa, H., Kosugi, S., Nakayama, S., Ono, A., Watanabe, A., Hashiguchi, M., Gondo, T., Ishigaki, G., Muguerza, M. and Shimizu, K. 2016. Sequencing and comparative analyses of the genomes of zoysiagrasses. DNA Research, 23(2), pp.171-180. | http://dx.doi.org/10.1093/dnares/dsw006 |
| Poaceae | *Zea mays* | 82.48 | 75.52 | 26.55 | 48.43 | 0.80 | 5.39 | Jia, J., Zhao, S., Kong, X., Li, Y., Zhao, G., He, W., Appels, R., Pfeifer, M., Tao, Y., Zhang, X. and Jing, R. 2013. Aegilops tauschii draft genome sequence reveals a gene repertoire for wheat adaptation. Nature, 496(7443), pp.91-95. | http://dx.doi.org/10.1038/nature12028 |
| Poaceae | *Zea mays* | 85.00 | 74.60 | 23.70 | 46.40 | 1.00 | 8.60 | Schnable, P.S., Ware, D., Fulton, R.S., Stein, J.C., Wei, F., Pasternak, S., Liang, C., Zhang, J., Fulton, L., Graves, T.A. and Minx, P. 2009. The B73 maize genome: complexity, diversity, and dynamics. science, 326(5956), pp.1112-1115. | http://dx.doi.org/10.1126/science.1178534 |
| Poaceae | *Zea mays* | 64.00 | 59.98 | 18.30 | 34.88 | 0.01 | 4.01 | Jiao, Y., Peluso, P., Shi, J., Liang, T., Stitzer, M.C., Wang, B., Campbell, M.S., Stein, J.C., Wei, X., Chin, C.S. and Guill, K. 2017. Improved maize reference genome with single-molecule technologies. Nature, 546(7659), pp.524-527. | http://dx.doi.org/10.1038/nature22971 |
| Poaceae | *Pharus latifolius* | 78.86 | 64.30 | 7.60 | 46.70 | 1.32 | 13.12 | Ma, P.F., Liu, Y.L., Jin, G.H., Liu, J.X., Wu, H., He, J., Guo, Z.H. and Li, D.Z. 2021. The Pharus latifolius genome bridges the gap of early grass evolution. The Plant Cell, 33(4), pp.846-864. | http://dx.doi.org/10.1093/plcell/koab015 |
| Poaceae | *Aegilops longissima* | 82.50 | 70.50 | 15.50 | 33.80 | 0.30 | 11.10 | Avni, R., Lux, T., Minz‐Dub, A., Millet, E., Sela, H., Distelfeld, A., Deek, J., Yu, G., Steuernagel, B., Pozniak, C. and Ens, J. 2022. Genome sequences of three Aegilops species of the section Sitopsis reveal phylogenetic relationships and provide resources for wheat improvement. The Plant Journal, 110(1), pp.179-192. | http://dx.doi.org/10.1111/tpj.15664 |
| Poaceae | *Aegilops sharonensis* | 82.30 | 70.40 | 15.50 | 33.70 | 0.30 | 11.10 | Avni, R., Lux, T., Minz‐Dub, A., Millet, E., Sela, H., Distelfeld, A., Deek, J., Yu, G., Steuernagel, B., Pozniak, C. and Ens, J. 2022. Genome sequences of three Aegilops species of the section Sitopsis reveal phylogenetic relationships and provide resources for wheat improvement. The Plant Journal, 110(1), pp.179-192. | http://dx.doi.org/10.1111/tpj.15664 |
| Poaceae | *Aegilops speltoides* | 78.70 | 69.40 | 18.30 | 30.80 | 0.50 | 8.30 | Avni, R., Lux, T., Minz‐Dub, A., Millet, E., Sela, H., Distelfeld, A., Deek, J., Yu, G., Steuernagel, B., Pozniak, C. and Ens, J. 2022. Genome sequences of three Aegilops species of the section Sitopsis reveal phylogenetic relationships and provide resources for wheat improvement. The Plant Journal, 110(1), pp.179-192. | http://dx.doi.org/10.1111/tpj.15664 |
| Poaceae | *Aegilops tauschii* | 80.50 | 63.80 | 15.80 | 26.90 | 0.40 | 15.90 | Avni, R., Lux, T., Minz‐Dub, A., Millet, E., Sela, H., Distelfeld, A., Deek, J., Yu, G., Steuernagel, B., Pozniak, C. and Ens, J. 2022. Genome sequences of three Aegilops species of the section Sitopsis reveal phylogenetic relationships and provide resources for wheat improvement. The Plant Journal, 110(1), pp.179-192. | http://dx.doi.org/10.1111/tpj.15664 |
| Poaceae | *Avena atlantica* | 82.97 | 66.69 | 17.26 | 47.89 | 0.92 | 6.00 | Maughan, P.J., Lee, R., Walstead, R., Vickerstaff, R.J., Fogarty, M.C., Brouwer, C.R., Reid, R.R., Jay, J.J., Bekele, W.A., Jackson, E.W. and Tinker, N.A. 2019. Genomic insights from the first chromosome-scale assemblies of oat (Avena spp.) diploid species. Bmc Biology, 17(1), pp.1-19. | http://dx.doi.org/10.1186/s12915-019-0712-y |
| Poaceae | *Avena eriantha* | 83.64 | NA | 13.84 | 48.44 | 1.20 | 7.40 | Maughan, P.J., Lee, R., Walstead, R., Vickerstaff, R.J., Fogarty, M.C., Brouwer, C.R., Reid, R.R., Jay, J.J., Bekele, W.A., Jackson, E.W. and Tinker, N.A. 2019. Genomic insights from the first chromosome-scale assemblies of oat (Avena spp.) diploid species. Bmc Biology, 17(1), pp.1-19. | http://dx.doi.org/10.1186/s12915-019-0712-y |
| Poaceae | *Avena sativa* | 86.95 | 70.63 | 5.74 | 33.03 | 4.78 | 8.96 | Peng, Y., Yan, H., Guo, L., Deng, C., Wang, C., Wang, Y., Kang, L., Zhou, P., Yu, K., Dong, X. and Liu, X. 2022. Reference genome assemblies reveal the origin and evolution of allohexaploid oat. Nature Genetics, 54(8), pp.1248-1258. | http://dx.doi.org/10.1038/s41588-022-01127-7 |
| Poaceae | *Brachypodium distachyon* | 37.48 | 18.38 | 4.46 | 13.77 | 2.94 | 5.33 | Jia, J., Zhao, S., Kong, X., Li, Y., Zhao, G., He, W., Appels, R., Pfeifer, M., Tao, Y., Zhang, X. and Jing, R. 2013. Aegilops tauschii draft genome sequence reveals a gene repertoire for wheat adaptation. Nature, 496(7443), pp.91-95. | http://dx.doi.org/10.1038/nature12028 |
| Poaceae | *Brachypodium distachyon* | 37.70 | 17.10 | 4.10 | 12.80 | 1.50 | 2.60 | Tanaka, H., Hirakawa, H., Kosugi, S., Nakayama, S., Ono, A., Watanabe, A., Hashiguchi, M., Gondo, T., Ishigaki, G., Muguerza, M. and Shimizu, K. 2016. Sequencing and comparative analyses of the genomes of zoysiagrasses. DNA Research, 23(2), pp.171-180. | http://dx.doi.org/10.1093/dnares/dsw006 |
| Poaceae | *Brachypodium distachyon* | 28.01 | 21.39 | 4.86 | 16.05 | 1.94 | 4.77 | DNA sequencing and assembly Barry Kerrie 5 Lucas Susan 5 Harmon-Smith Miranda 5 Lail Kathleen 5 Tice Hope 5 Schmutz (Leader) Jeremy 4 Grimwood Jane 4 McKenzie Neil 7 Bevan Michael W. michael. bevan@ bbsrc. ac. uk 7 k, Gene analysis and annotation Haberer Georg 16 Spannagl Manuel 16 Mayer (Leader) Klaus 16 Rattei Thomas 17 Mitros Therese 6 Rokhsar Dan 6 Lee Sang-Jik 18 Rose Jocelyn KC 18 Mueller Lukas A. 19 York Thomas L. 19 and Comparative genomics Salse (Leader) Jerome 27 Murat Florent 27 Abrouk Michael 27 Haberer Georg 16 Spannagl Manuel 16 Mayer Klaus 16 Bruggmann Remy 13 Messing Joachim 13 You Frank M. 8 Luo Ming-Cheng 8 Dvorak Jan 8, 2010. Genome sequencing and analysis of the model grass Brachypodium distachyon. Nature, 463(7282), pp.763-768. | http://dx.doi.org/10.1038/nature08747 |
| Poaceae | *Hordeum spontaneum* | 86.50 | 74.30 | 19.30 | 27.00 | NA | 12.30 | Zhang, W., Tan, C., Hu, H., Pan, R., Xiao, Y., Ouyang, K., Zhou, G., Jia, Y., Zhang, X.Q., Hill, C.B. and Wang, P. 2023. Genome architecture and diverged selection shaping pattern of genomic differentiation in wild barley. Plant biotechnology journal, 21(1), pp.46-62. | http://dx.doi.org/10.1111/pbi.13917 |
| Poaceae | *Poa annua* | 74.50 | 39.23 | 12.35 | 25.88 | 2.12 | 3.33 | Benson, C.W., Sheltra, M.R., Maughan, P.J., Jellen, E.N., Robbins, M.D., Bushman, B.S., Patterson, E.L., Hall, N.D. and Huff, D.R. 2023. Homoeologous evolution of the allotetraploid genome of Poa annua L. BMC Genomics, 24(1), p.350. | http://dx.doi.org/10.1186/s12864-023-09456-5 |
| Poaceae | *Poa infirma* | 81.20 | 53.00 | 14.53 | 29.03 | 1.87 | 2.98 | Benson, C.W., Sheltra, M.R., Maughan, P.J., Jellen, E.N., Robbins, M.D., Bushman, B.S., Patterson, E.L., Hall, N.D. and Huff, D.R. 2023. Homoeologous evolution of the allotetraploid genome of Poa annua L. BMC Genomics, 24(1), p.350. | http://dx.doi.org/10.1186/s12864-023-09456-5 |
| Poaceae | *Secale cereale* | 90.31 | 76.20 | 15.30 | 54.90 | 1.16 | 11.80 | Li, G., Wang, L., Yang, J., He, H., Jin, H., Li, X., Ren, T., Ren, Z., Li, F., Han, X. and Zhao, X. 2021. A high-quality genome assembly highlights rye genomic characteristics and agronomically important genes. Nature genetics, 53(4), pp.574-584. | http://dx.doi.org/10.1038/s41588-021-00808-z |
| Poaceae | *Triticum aestivum* | 82.30 | 68.90 | 16.70 | 32.40 | 0.40 | 12.50 | Avni, R., Lux, T., Minz‐Dub, A., Millet, E., Sela, H., Distelfeld, A., Deek, J., Yu, G., Steuernagel, B., Pozniak, C. and Ens, J. 2022. Genome sequences of three Aegilops species of the section Sitopsis reveal phylogenetic relationships and provide resources for wheat improvement. The Plant Journal, 110(1), pp.179-192. | http://dx.doi.org/10.1111/tpj.15664 |
| Poaceae | *Triticum aestivum* | 81.70 | 66.60 | 10.20 | 44.10 | NA | 14.50 | International Wheat Genome Sequencing Consortium (IWGSC), Mayer, K.F., Rogers, J., Doležel, J., Pozniak, C., Eversole, K., Feuillet, C., Gill, B., Friebe, B., Lukaszewski, A.J. and Sourdille, P. 2014. A chromosome-based draft sequence of the hexaploid bread wheat (Triticum aestivum) genome. Science, 345(6194), p.1251788. | http://dx.doi.org/10.1126/science.1251788 |
| Poaceae | *Triticum aestivum* | 81.20 | 63.00 | 10.30 | 45.50 | NA | 17.50 | International Wheat Genome Sequencing Consortium (IWGSC), Mayer, K.F., Rogers, J., Doležel, J., Pozniak, C., Eversole, K., Feuillet, C., Gill, B., Friebe, B., Lukaszewski, A.J. and Sourdille, P. 2014. A chromosome-based draft sequence of the hexaploid bread wheat (Triticum aestivum) genome. Science, 345(6194), p.1251788. | http://dx.doi.org/10.1126/science.1251788 |
| Poaceae | *Triticum aestivum* | 79.90 | 59.90 | 8.20 | 39.80 | NA | 19.30 | International Wheat Genome Sequencing Consortium (IWGSC), Mayer, K.F., Rogers, J., Doležel, J., Pozniak, C., Eversole, K., Feuillet, C., Gill, B., Friebe, B., Lukaszewski, A.J. and Sourdille, P. 2014. A chromosome-based draft sequence of the hexaploid bread wheat (Triticum aestivum) genome. Science, 345(6194), p.1251788. | http://dx.doi.org/10.1126/science.1251788 |
| Poaceae | *Triticum urartu* | 66.88 | 46.66 | 9.89 | 36.57 | 2.34 | 9.77 | Ling, H.Q., Zhao, S., Liu, D., Wang, J., Sun, H., Zhang, C., Fan, H., Li, D., Dong, L., Tao, Y. and Gao, C. 2013. Draft genome of the wheat A-genome progenitor Triticum urartu. Nature, 496(7443), pp.87-90. | http://dx.doi.org/10.1038/nature11997 |
| Typhaceae | *Sparganium stoloniferum* | 61.19 | 55.71 | NA | NA | 0.77 | 1.55 | Zou, Y., Wei, Z., Xiao, K., Wu, Z. and Xu, X. 2023. Genomic analysis of the emergent aquatic plant Sparganium stoloniferum provides insights into its clonality, local adaptation and demographic history. Molecular Ecology Resources. | <https://onlinelibrary.wiley.com/doi/10.1111/1755-0998.13850> |
| Typhaceae | *Typha angustifolia* | 27.60 | 15.65 | 3.32 | 8.93 | NA | NA | Liao, Y., Zhao, S., Zhang, W., Zhao, P., Lu, B., Moody, M.L., Tan, N. and Chen, L. 2023. Chromosome-level genome and high nitrogen stress response of the widespread and ecologically important wetland plant Typha angustifolia. Frontiers in Plant Science, 14, p.1138498. | <https://www.frontiersin.org/articles/10.3389/fpls.2023.1138498/full> |
| Typhaceae | *Typha latifolia* | 43.84 | 15.35 | NA | NA | 1.22 | 1.30 | Widanagama, S.D., Freeland, J.R., Xu, X. and Shafer, A.B. 2022. Genome assembly, annotation, and comparative analysis of the cattail Typha latifolia. G3, 12(2), p.jkab401. | <https://academic.oup.com/g3journal/article/12/2/jkab401/6433155> |
| Alpinioideae | *Alpinia oxyphylla* | 88.06 | 61.70 | NA | NA | 1.04 | 2.44 | Pan, K., Dai, S., Tian, J., Zhang, J., Liu, J., Li, M., Li, S., Zhang, S. and Gao, B. 2023. Chromosome-level genome and multi-omics analyses provide insights into the geo-herbalism properties of Alpinia oxyphylla. Frontiers in Plant Science, 14, p.1161257. | <https://www.frontiersin.org/articles/10.3389/fpls.2023.1161257/full> |
| Alpinioideae | *Elettaria cardamomum* | 71.15 | 46.00 | 36.00 | 9.00 | NA | 0.97 | Gaikwad, A.B., Kumari, R., Yadav, S., Rangan, P., Wankhede, D.P. and Bhat, K.V. 2023. Small cardamom genome: development and utilization of microsatellite markers from a draft genome sequence of Elettaria cardamomum Maton. Frontiers in Plant Science, 14, p.1161499. | <https://www.frontiersin.org/articles/10.3389/fpls.2023.1161499/full> |
| Alpinioideae | *Lanxangia tsao-ko* | 78.90 | 62.50 | 44.44 | 16.69 | NA | NA | Li, P., Bai, G., He, J., Liu, B., Long, J., Morcol, T., Peng, W., Quan, F., Luan, X., Wang, Z. and Zhao, Y. 2022. Chromosome-level genome assembly of Amomum tsao-ko provides insights into the biosynthesis of flavor compounds. Horticulture Research, 9. | <https://academic.oup.com/hr/article/doi/10.1093/hr/uhac211/6705571> |
| Alpinioideae | *Lanxangia tsao-ko* | 89.15 | 76.28 | 35.55 | 19.16 | 0.91 | 1.93 | Sun, F., Yan, C., Lv, Y., Pu, Z., Liao, Z., Guo, W. and Dai, M. 2022. Genome Sequencing of Amomum tsao-ko Provides Novel Insight Into Its Volatile Component Biosynthesis. Frontiers in Plant Science, 13. | <https://www.frontiersin.org/articles/10.3389/fpls.2022.904178/full> |
| Alpinioideae | *Wurfbainia longiligularis* | 85.27 | 76.32 | 51.44 | 21.30 | NA | 6.85 | Yang, P., Ling, X.Y., Zhou, X.F., Chen, Y.X., Wang, T.T., Lin, X.J., Zhao, Y.Y., Ye, Y.S., Huang, L.X., Sun, Y.W. and Qi, Y.X. 2023. Comparing genomes of Fructus Amomi-producing species reveals genetic basis of volatile terpenoid divergence. Plant Physiology, p.kiad400. | <https://academic.oup.com/plphys/advance-article/doi/10.1093/plphys/kiad400/7222147> |
| Alpinioideae | *Wurfbainia villosa* | 87.14 | 78.94 | 52.92 | 23.80 | NA | 6.40 | Yang, P., Ling, X.Y., Zhou, X.F., Chen, Y.X., Wang, T.T., Lin, X.J., Zhao, Y.Y., Ye, Y.S., Huang, L.X., Sun, Y.W. and Qi, Y.X. 2023. Comparing genomes of Fructus Amomi-producing species reveals genetic basis of volatile terpenoid divergence. Plant Physiology, p.kiad400. | <https://academic.oup.com/plphys/advance-article/doi/10.1093/plphys/kiad400/7222151> |
| Alpinioideae | *Wurfbainia villosa* | 87.23 | 78.26 | NA | NA | 0.73 | 2.59 | Yang, P., Zhao, H.Y., Wei, J.S., Zhao, Y.Y., Lin, X.J., Su, J., Li, F.P., Li, M., Ma, D.M., Tan, X.K. and Liang, H.L. 2022. Chromosome‐level genome assembly and functional characterization of terpene synthases provide insights into the volatile terpenoid biosynthesis of Wurfbainia villosa. The Plant Journal, 112(3), pp.630-645. | <https://onlinelibrary.wiley.com/doi/10.1111/tpj.15968> |
| Zingiberoideae | *Boesenbergia rotunda* | 72.51 | 67.16 | NA | NA | NA | 3.29 | Taheri, S., Teo, C.H., Heslop-Harrison, J.S., Schwarzacher, T., Tan, Y.S., Wee, W.Y., Khalid, N., Biswas, M.K., Mutha, N.V., Mohd-Yusuf, Y. and Gan, H.M. 2022. Genome assembly and analysis of the flavonoid and phenylpropanoid biosynthetic pathways in Fingerroot ginger (Boesenbergia rotunda). International Journal of Molecular Sciences, 23(13), p.7269. | <https://www.mdpi.com/1422-0067/23/13/7269> |
| Zingiberoideae | *Curcuma alismatifolia* | 72.31 | 62.92 | 33.86 | 27.82 | NA | 8.76 | Yang, P., Ling, X.Y., Zhou, X.F., Chen, Y.X., Wang, T.T., Lin, X.J., Zhao, Y.Y., Ye, Y.S., Huang, L.X., Sun, Y.W. and Qi, Y.X. 2023. Comparing genomes of Fructus Amomi-producing species reveals genetic basis of volatile terpenoid divergence. Plant Physiology, p.kiad400. | <https://academic.oup.com/plphys/advance-article/doi/10.1093/plphys/kiad400/7222148> |
| Zingiberoideae | *Curcuma alismatifolia* | 79.28 | 68.45 | NA | NA | 1.24 | 2.46 | Dong, Q., Zou, Q.C., Mao, L.H., Tian, D.Q., Hu, W., Cao, X.R. and Ding, H.Q. 2022. The Chromosome-Scale Assembly of the Curcuma alismatifolia Genome Provides Insight Into Anthocyanin and Terpenoid Biosynthesis. Frontiers in Plant Science, 13, p.899588. | <https://www.frontiersin.org/articles/10.3389/fpls.2022.899588/full> |
| Zingiberoideae | *Curcuma alismatifolia* | NA | 52.60 | 31.79 | 20.81 | NA | NA | Liao, X., Ye, Y., Zhang, X., Peng, D., Hou, M., Fu, G., Tan, J., Zhao, J., Jiang, R., Xu, Y. and Liu, J. 2022. The genomic and bulked segregant analysis of Curcuma alismatifolia revealed its diverse bract pigmentation. Abiotech, 3(3), pp.178-196. | <https://link.springer.com/article/10.1007/s42994-022-00081-6> |
| Zingiberoideae | *Curcuma longa* | 64.16 | 27.37 | 17.19 | 9.42 | 1.13 | 2.26 | Chakraborty, A., Mahajan, S., Jaiswal, S.K. and Sharma, V.K. 2021. Genome sequencing of turmeric provides evolutionary insights into its medicinal properties. Communications Biology, 4(1), p.1193. | <https://www.nature.com/articles/s42003-021-02720-y> |
| Zingiberoideae | *Curcuma longa* | 70.00 | 50.00 | 31.09 | 21.40 | 0.64 | 8.40 | Yin, Y., Xie, X., Zhou, L., Yin, X., Guo, S., Zhou, X., Li, Q., Shi, X., Peng, C. and Gao, J. 2022. A chromosome-scale genome assembly of turmeric provides insights into curcumin biosynthesis and tuber formation mechanism. Frontiers in Plant Science, 13, p.3685. | <https://www.frontiersin.org/articles/10.3389/fpls.2022.1003835/full> |
| Zingiberoideae | *Zingiber officinale* | 77.81 | 69.92 | 35.95 | 31.43 | NA | 6.31 | Yang, P., Ling, X.Y., Zhou, X.F., Chen, Y.X., Wang, T.T., Lin, X.J., Zhao, Y.Y., Ye, Y.S., Huang, L.X., Sun, Y.W. and Qi, Y.X. 2023. Comparing genomes of Fructus Amomi-producing species reveals genetic basis of volatile terpenoid divergence. Plant Physiology, p.kiad400. | <https://academic.oup.com/plphys/advance-article/doi/10.1093/plphys/kiad400/7222147> |
| Zingiberoideae | *Zingiber officinale* | 81.70 | 56.57 | 33.66 | 21.69 | 1.29 | 4.30 | Cheng, S.P., Jia, K.H., Liu, H., Zhang, R.G., Li, Z.C., Zhou, S.S., Shi, T.L., Ma, A.C., Yu, C.W., Gao, C. and Cao, G.L. 2021. Haplotype-resolved genome assembly and allele-specific gene expression in cultivated ginger. Horticulture Research, 8. | <https://academic.oup.com/hr/article/doi/10.1038/s41438-021-00599-8/6446763> |
| Zingiberoideae | *Zingiber officinale* | 56.70 | 61.06 | NA | NA | 1.05 | 1.85 | Li, H.L., Wu, L., Dong, Z., Jiang, Y., Jiang, S., Xing, H., Li, Q., Liu, G., Tian, S., Wu, Z. and Wu, B. 2021. Haplotype-resolved genome of diploid ginger (Zingiber officinale) and its unique gingerol biosynthetic pathway. Horticulture Research, 8. | <https://academic.oup.com/hr/article/doi/10.1038/s41438-021-00627-7/6446767> |

**Supplementary Table 5.** The list of FISH probes targeting the GAG domains of SIRE and Tekay elements in the genus *Amomum*. Clades inferred from ML phylogeny refer to Supplementary Figure 1 (SIRE) and Supplementary Figure 2 (Tekay).

| **Marker** | **Type of probe** | | **Element (domain)** | | | **Clades inferred from ML phylogeny** | **Sequence** | | | | **Probe GC content (%)** |  |
| --- | --- | --- | --- | --- | --- | --- | --- | --- | --- | --- | --- | --- |
|  | |  | |  |  | | **Forward primer** | **Sequence** | **Reverse primer** | **Sequence** | **Theoretical amplicon (bp)** | |
| sire_pcr_cl_II | | PCR primers | | SIRE - GAG | II | | sire_pcr_cl_II_F | TCCATTCAGCAGTGCTCAGG | sire_pcr_cl_II_R | AACATACCTGGCGAGATCCC | 226 | |
| sire_pcr_cl_VI | | PCR primers | | SIRE - GAG | VI | | sire_pcr_cl_VI_F | CGGTCTAACCAAGGAGGAGC | sire_pcr_cl_VI_R | GTGCAGCCCATTCAGGATCT | 214 | |
| tekay_pcr_cl_I | | PCR primers | | Tekay - GAG | I | | tekay_pcr_cl_I_F | ATGCTCAGGCCTGGTTCAAG | tekay_pcr_cl_I_R | GTTGCCTTGACGAAACTCCG | 251 | |
| tekay_pcr_cl_III | | PCR primers | | Tekay - GAG | III | | tekay_pcr_cl_III_F | ATCATTTCCGGGATCAGGCC | tekay_pcr_cl_III_R | CCCTGCTTTAGGCCCAGAAA | 263 | |

**Supplementary Table 6.** Phylogenetically significant repeat clusters. Seventy-five phylogenetically significant clusters, along with their Pagel’s lambda and p values, quantities in clades A+B+C versus clade D, and trends marked as increasing (+) or decreasing (-). These clusters are sourced from the RepeatExplorer comparative analysis.

| **superfamily/group** | **lineage** | **λ** | **p-value** | **amount in A+B+C (Mb)** | **amount in D (Mb)** | **trend** | **cluster no.** |
| --- | --- | --- | --- | --- | --- | --- | --- |
| *Ty1-Copia* | Ale | 1 | 0.003 | 155 | 1386 | + | 89 |
| - | other | 1 | 0.001 | 17 | 1086 | + | 112 |
| - | other | 1 | 0.003 | 164 | 479 | + | 139 |
| - | other | 1 | 0.006 | 518 | 243 | - | 141 |
| - | other | 0.967 | 0.036 | 428 | 158 | - | 154 |
| - | other | 1 | 0.009 | 406 | 43 | - | 167 |
| - | other | 1 | 0.008 | 270 | 102 | - | 174 |
| - | other | 1 | 0.038 | 15 | 178 | + | 193 |
| - | other | 1 | 0.001 | 169 | 59 | - | 200 |
| - | other | 1 | 0.022 | 4 | 116 | + | 208 |
| - | other | 1 | 0.016 | 81 | 40 | - | 221 |
| - | other | 1 | 0.008 | 81 | 28 | - | 225 |
| - | other | 1 | 0.006 | 5 | 66 | + | 232 |
| - | other | 1 | 0.021 | 10 | 51 | + | 244 |
| - | other | 1 | 0.004 | 95 | 1 | - | 247 |
| *Ty1-Copia* | Angela | 1 | 0.007 | 5121 | 1009 | - | 21 |
| *Ty1-Copia* | Angela | 1 | 0.027 | 185 | 77 | - | 24 |
| *Ty1-Copia* | Angela | 1 | 0.002 | 3796 | 865 | - | 32 |
| *Ty1-Copia* | Angela | 1 | 0.008 | 3929 | 698 | - | 35 |
| *Ty1-Copia* | Angela | 1 | 0.01 | 2875 | 924 | - | 44 |
| *Ty1-Copia* | Angela | 1 | 0.007 | 2963 | 815 | - | 45 |
| *Ty1-Copia* | Angela | 1 | 0.006 | 2854 | 557 | - | 53 |
| *Ty1-Copia* | Angela | 1 | 0.007 | 2112 | 627 | - | 70 |
| *Ty1-Copia* | Angela | 1 | 0.009 | 2232 | 437 | - | 73 |
| *Ty1-Copia* | Angela | 1 | 0.006 | 1970 | 565 | - | 75 |
| *Ty1-Copia* | Angela | 1 | 0.009 | 2209 | 317 | - | 81 |
| *Ty1-Copia* | Angela | 1 | 0.005 | 1842 | 367 | - | 92 |
| *Ty1-Copia* | Angela | 1 | 0.007 | 1827 | 368 | - | 93 |
| *Ty1-Copia* | Angela | 1 | 0.023 | 1610 | 363 | - | 102 |
| *Ty1-Copia* | Angela | 1 | 0.006 | 1402 | 446 | - | 104 |
| *Ty1-Copia* | Angela | 1 | 0.006 | 1182 | 273 | - | 119 |
| *Ty1-Copia* | Angela | 1 | 0.007 | 314 | 58 | - | 179 |
| *Ty3-Gypsy* | Athila | 1 | 0.016 | 1151 | 646 | - | 100 |
| *Ty3-Gypsy* | Athila | 1 | 0.003 | 1018 | 353 | - | 121 |
| - | LINE | 1 | 0.008 | 741 | 1918 | + | 50 |
| - | LINE | 1 | 0.017 | 168 | 357 | + | 149 |
| - | LINE | 0.86 | 0.046 | 38 | 133 | + | 201 |
| - | unclassified LTR | 1 | 0.008 | 9448 | 4329 | - | 1 |
| - | unclassified LTR | 1 | 0.003 | 9422 | 3340 | - | 2 |
| - | unclassified LTR | 1 | 0.014 | 50 | 5643 | + | 12 |
| - | Unclassified LTR | 1 | 0.004 | 4472 | 1838 | - | 18 |
| - | Unclassified LTR | 0.937 | 0.044 | 416 | 3219 | + | 25 |
| - | Unclassified LTR | 1 | 0.001 | 1943 | 757 | - | 65 |
| - | Unclassified LTR | 1 | 0.014 | 1662 | 113 | - | 113 |
| DNA transposons | MuDR_Mutator | 0.87 | 0.043 | 433 | 1282 | + | 84 |
| DNA transposons | MuDR_Mutator | 1 | 0.049 | 736 | 407 | - | 129 |
| *Ty3-Gypsy* | Retand | 1 | 0.033 | 3505 | 1783 | - | 22 |
| *Ty3-Gypsy* | Retand | 1 | 0.003 | 253 | 1284 | + | 91 |
| *Ty3-Gypsy* | Retand | 1 | 0.01 | 201 | 513 | + | 135 |
| *Ty1-Copia* | SIRE | 1 | 0.002 | 634 | 6718 | + | 4 |
| *Ty1-Copia* | SIRE | 1 | 0.003 | 333 | 5273 | + | 13 |
| *Ty1-Copia* | SIRE | 1 | 0.008 | 1348 | 4592 | + | 15 |
| *Ty1-Copia* | SIRE | 1 | 0.02 | 3896 | 2235 | - | 17 |
| *Ty1-Copia* | SIRE | 1 | 0.021 | 2959 | 1572 | - | 29 |
| *Ty1-Copia* | SIRE | 1 | 0.03 | 1272 | 2348 | + | 31 |
| *Ty1-Copia* | SIRE | 1 | 0.044 | 4 | 2969 | + | 34 |
| *Ty1-Copia* | SIRE | 1 | 0.028 | 1193 | 2245 | + | 36 |
| *Ty1-Copia* | SIRE | 1 | 0.031 | 2628 | 1292 | - | 37 |
| *Ty1-Copia* | SIRE | 0.963 | 0.029 | 871 | 1685 | + | 56 |
| *Ty1-Copia* | SIRE | 1 | 0.01 | 32 | 2145 | + | 57 |
| *Ty1-Copia* | SIRE | 1 | 0.014 | 2164 | 860 | - | 58 |
| *Ty1-Copia* | SIRE | 0.963 | 0.034 | 785 | 1538 | + | 61 |
| *Ty1-Copia* | SIRE | 1 | 0.024 | 1982 | 729 | - | 67 |
| *Ty1-Copia* | SIRE | 1 | 0.001 | 2400 | 485 | - | 68 |
| *Ty1-Copia* | SIRE | 1 | 0.006 | 1815 | 800 | - | 69 |
| *Ty1-Copia* | SIRE | 1 | 0.031 | 1292 | 640 | - | 94 |
| *Ty1-Copia* | SIRE | 1 | 0.031 | 1174 | 649 | - | 98 |
| *Ty1-Copia* | SIRE | 1 | 0.001 | 1237 | 410 | - | 108 |
| *Ty1-Copia* | SIRE | 1 | 0.002 | 1165 | 445 | - | 111 |
| *Ty1-Copia* | SIRE | 1 | 0.002 | 255 | 795 | + | 120 |
| *Ty1-Copia* | SIRE | 1 | 0.001 | 435 | 143 | - | 156 |
| *Ty3-Gypsy* | Tekay | 1 | 0.011 | 1539 | 5618 | + | 7 |
| *Ty3-Gypsy* | Tekay | 1 | 0.005 | 272 | 824 | + | 115 |
| *Ty3-Gypsy* | Tekay | 1 | 0.023 | 20 | 715 | + | 131 |
| *Ty3-Gypsy* | All | 1 | 0.016 | 1309 | 173 | - | 123 |
